# Model-based economic evaluation of the effectiveness of “‘Hypos’ can strike twice”, a leaflet-based ambulance clinician referral intervention to prevent recurrent hypoglycaemia

**DOI:** 10.1371/journal.pone.0282987

**Published:** 2023-03-16

**Authors:** Murray D. Smith, Colin Ridyard, Vanessa Botan, Amanda Brewster, Sally Dunmore, June James, Kamlesh Khunti, Despina Laparidou, Graham Law, Pauline Mountain, Leon Roberts, Elise Rowan, Robert Spaight, Keith Spurr, Aloysius N. Siriwardena

**Affiliations:** 1 Community and Health Research Unit, University of Lincoln, Lincoln, United Kingdom; 2 Clinical Audit and Research Unit, East Midlands Ambulance Service NHS Trust, Nottingham, United Kingdom; 3 Patient and Public Contributor, Lincoln, United Kingdom; 4 University Hospitals of Leicester NHS Trust, Leicester, United Kingdom; 5 Leicester Diabetes Centre, University of Leicester, Leicester, United Kingdom; University of Huddersfield, UNITED KINGDOM

## Abstract

“‘Hypos’ can strike twice” (HS2) is a pragmatic, leaflet-based referral intervention designed for administration by clinicians of the emergency medical services (EMS) to people they have attended and successfully treated for hypoglycaemia. Its main purpose is to encourage the recipient to engage with their general practitioner or diabetic nurse in order that improvements in medical management of their diabetes may be made, thereby reducing their risk of recurrent hypoglycaemia. Herein we build a de novo economic model for purposes of incremental analyses to compare, in 2018–19 prices, HS2 against standard care for recurrent hypoglycaemia in the fortnight following the initial attack from the perspective of the UK National Health Service (NHS). We found that per patient NHS costs incurred by people receiving the HS2 intervention over the fortnight following an initial hypoglycaemia average £49.79, and under standard care costs average £40.50. Target patient benefit assessed over that same period finds the probability of no recurrence of hypoglycaemia averaging 42.4% under HS2 and 39.4% under standard care, a 7.6% reduction in relative risk. We find that implementing HS2 will cost the NHS an additional £309.36 per episode of recurrent hypoglycaemia avoided. Contrary to the favourable support offered in Botan et al., we conclude that in its current form the HS2 intervention is not a cost-effective use of NHS resources when compared to standard NHS care in reducing the risk of hypoglycaemia recurring within a fortnight of an initial attack that was resolved at-scene by EMS ambulance clinicians.

## Introduction

Hypoglycaemia, a common adverse complication of diabetes treatment [1], is often managed by patients themselves or their relatives, but severe hypoglycaemia (defined as cognitive impairment severe enough to require third-party assistance to take corrective action [2]) frequently requires attendance of an emergency medical services (EMS) ambulance. EMS clinicians may successfully resolve the hypoglycaemia at-scene, equally the patient may need to be conveyed to an Emergency Department (ED) for further treatment and possible hospital admission [3].

A systematic review highlighted that those with hypoglycaemia attended by ambulance services frequently have recurrent episodes, do not attend primary care when advised to do so by ambulance staff, but often require changes in therapy to prevent further episodes. It concluded with a recommendation to develop and evaluate community referral pathways for hypoglycaemia [4].

The “‘Hypos’ can strike twice” (HS2) leaflet-based intervention is a pragmatic referral activity administered by EMS clinicians to people they have attended and successfully treated for hypoglycaemia. Its main purpose is to encourage the recipient to engage with their general practitioner (GP) or diabetic nurse in order that improvements in medical management of their diabetes may be made, and in so doing reduce their risk of recurrent hypoglycaemia. Its secondary purpose is to inform the recipient about how they may better manage their diabetes through lifestyle change. A further benefit expected from the intervention is a reduction in the EMS attendance rate for recurrent hypoglycaemia.

The intervention was subject to trial in the East Midlands Ambulance Service NHS Trust for a period of 26 months. The trial’s primary outcome measured repeat EMS attendance for recurrent hypoglycaemia within a fortnight of the initial attack.

Motivating the need for an economic evaluation of the HS2 intervention is its low cost, where this comprises booklet production plus the EMS ambulance crew cost arising from the length of time taken to administer it. In addition, Botan et al. [5] and Laparidou et al. [6] have published reports on the HS2 trial, where the former used statistical modelling to conclude, that by implementing HS2, ambulance clinicians could significantly prevent future attendances for recurrent hypoglycaemic events as well as asserting that it reduces health costs. Those findings, which provide further motivation to evaluate the economic performance of the HS2 intervention, were, however, predicated on analyses of outcomes in which longer-term time horizons further than 90 days beyond the initial attack were measured.

For this evaluation we build a de novo economic model for recurrent hypoglycaemia focussing on the fortnight following the initial attack, as per the trial’s designated primary outcome. Wherever possible data gathered from the HS2 trial were used, alongside of which inputs from the project’s Expert Patient and Clinician Group (EPCG) were especially useful in model design as well as its parameterisation. We then use the model to conduct incremental economic analyses to examine whether the HS2 intervention added to standard care is cost-effective versus standard care alone. The model is then extended in repeated fortnightly cycles to estimate the probability that HS2 may reduce health costs compared to those accrued under standard care alone.

## Materials and methods

### Trial details

The HS2 trial was registered with ClinicalTrials.gov: NCT04243200 on 27 January 2020 [7]. The trial protocol “Ambu-HS2 Protocol v1.1” is in S2 File. Ethics approval was obtained from Yorkshire and The Humber—Leeds West Research Ethics Committee, reference 20/YH/0082 (IRAS ID 276438), 2 March 2020 (see https://www.hra.nhs.uk/planning-and-improving-research/application-summaries/research-summaries/ambulance-hypos-can-strike-twice-ambu-hs2-study-version-10/) and Research and Development approval from East Midlands Ambulance Service NHS Trust. The sponsor was the University of Lincoln 191202. The trial was funded by the National Institute for Health Research, Applied Research Collaboration (East Midlands). Data are from human subjects, but we did not obtain informed consent because the design involved use of routine anonymised data.

### Target population

The target population is people attended by EMS clinicians where the patient’s hypoglycaemia is resolved at the scene of the incident. Annually, approximately 18,000 people would be eligible to receive the intervention if it was made available across England and Wales. (In financial year 2009–10, approximately 0.6% of emergency calls attended by the East Midlands Ambulance Service NHS Trust were for severe hypoglycaemia [8]. Assuming this same rate prevails nationwide in financial year 2018–19, then across a total of 7.9m ambulance attendances across England and Wales [9], with adjustments for 5% repeated attendances within 2-weeks and a conveyance rate to ED of 60% (HS2 trial), then the annual number of patients eligible to receive the HS2 intervention is estimated to be approximately 18000 = 7.9mx0.006(1–0.05)(1–0.6).)

### Intervention

The HS2 intervention is an at-scene EMS referral activity undertaken after the patient’s hypoglycaemia has been successfully resolved and prior to their discharge at-scene; it is additive to standard care. The patient is issued the HS2 booklet within which EMS clinicians record vital details and measurements, before and after treatment, and organise, during business hours, an appointment for the patient to consult their GP. Not only is the patient encouraged to read the booklet, but they are asked to take the booklet with them to their consultation as a record of their recent hypoglycaemia. The HS2 intervention is regarded as successful if the patient attends the consultation and partially successful if the patient only reads the booklet.

### Comparator

The comparator is standard EMS care and assumes no at-scene referral activity is undertaken on the eligible target population of patients by EMS clinicians; namely, those patients that have been discharged at-scene because their hypoglycaemia has been successfully resolved.

### Time horizon

The time horizon of analysis was governed by the primary outcome of the HS2 trial; namely, recurring hypoglycaemia in the fortnight following the initial attack. Further analysis in line with the secondary outcomes of the HS2 trial involved longer-term modelling in which the time horizon was extended up to, but not beyond 90 days of the initial attack.

### Outcomes and analyses

First, as the baseline case we carry out a cost-effectiveness analysis in terms of whether or not hypoglycaemia recurs in the target population. Second, given literature-sourced societal utility weights for severity of hypoglycaemia [10], the latter distinguishes between severe and non-severe hypoglycaemia (denoted respectively “HypoS” and “HypoNS”), we conduct a cost-utility analysis in terms of quality adjusted life years (QALY). Third, the HS2 intervention may be regarded as succeeding if its recipient engages with their GP or diabetic nurse sooner rather than later. Over time increasingly more patients will consult their GP about their diabetes as part of routine diabetes care irrespective of whether or not they receive the HS2 intervention, driving to zero any difference in patient benefit between intervention and standard care. The issue then becomes whether the initial boost in GP consultation due to the HS2 intervention has been sufficient to save on costs to the NHS. To explore this, we carry out a cost minimisation analysis involving repeated fortnight-length cycles.

### Cost schedule

The costs of treating hypoglycaemia are evaluated from the perspective of the NHS and these may be incurred by a number of service providers: EMS, primary care, Integrated Urgent Care (IUC) and secondary care. The cost schedule is given in Table 1. The price year is 2018–19.

**Table 1:** Cost schedule.

Item	Cost (£)	Unit	Notes
111	7.33	Call	Call 111 for IUC. Cost assumed equivalent to call 999
999	7.33	Call	Call 999 for EMS. Source: National Schedule of NHS Costs 2018–19 [9], HRG code ASC1
ED1A	262.57	Attendance	Emergency medicine type 01 admitted. Average weighted by attendances. Source: National Schedule of NHS Costs 2018–19 [9], HRG codes VB(01–09)Z+VB11Z
ED1NA	170.66	Attendance	Emergency medicine type 01 not admitted. Average weighted by attendances. Source: National Schedule of NHS Costs 2018–19 [9], HRG codes VB(01–09)Z+VB11Z
ED2A	142.26	Attendance	Emergency medicine type 02 admitted. Average weighted by attendances. Source: National Schedule of NHS Costs 2018–19 [9], HRG codes VB(01–09)Z+VB11Z
ED2NA	109.92	Attendance	Emergency medicine type 02 not admitted. Average weighted by attendances. Source: National Schedule of NHS Costs 2018–19 [9], HRG codes VB(01–09)Z+VB11Z
ED3A	115.69	Attendance	Emergency medicine type 03 admitted. Average weighted by attendances. Source: National Schedule of NHS Costs 2018–19 [9], HRG codes VB(01–09)Z+VB11Z
ED3NA	73.57	Attendance	Emergency medicine type 03 not admitted. Average weighted by attendances. Source: National Schedule of NHS Costs 2018–19 [9], HRG codes VB(01–09)Z+VB11Z
ED4A	44.17	Attendance	Emergency medicine type 04 admitted. Average weighted by attendances. Source: National Schedule of NHS Costs 2018–19 [9], HRG codes VB(01–09)Z+VB11Z
ED4NA	45.71	Attendance	Emergency medicine type 04 not admitted. Average weighted by attendances. Source: National Schedule of NHS Costs 2018–19 [9], HRG codes VB(01–09)Z+VB11Z
GP	39.00	Consultation	General practitioner, family doctor consultation. Source: Curtis and Burns [11]
HA	1215.74	Episode	Hospital admission. Episode weighted average of non-elective short- and long-stay of HRG codes KA06x, KA08x, KB01x, WH04x then combined with respective weights 1/32, 3/58, 1, 1/136 scaled to sum to 1.
HS2 administration	2.55	Minute	Dual crewed ambulance. Source: Siriwardena et al. [12; Table 14, column 6]
HS2 booklet	1.60	Booklet	Cost of production of 16-page booklet.
HTR	47.49	Patient	EMS hear, treat and/or refer then remote discharge. Source: National Schedule of NHS Costs 2018–19 [9], HRG code ASH1
STC	257.34	Incident	EMS see, treat and convey to an ED for further health care. Source: National Schedule of NHS Costs 2018–19 [9], HRG code ASS02
STR	209.38	Incident	EMS see, treat and/or refer then discharge at scene. Source: National Schedule of NHS Costs 2018–19 [9], HRG code ASS01

### Economic model

**Fig 1:**
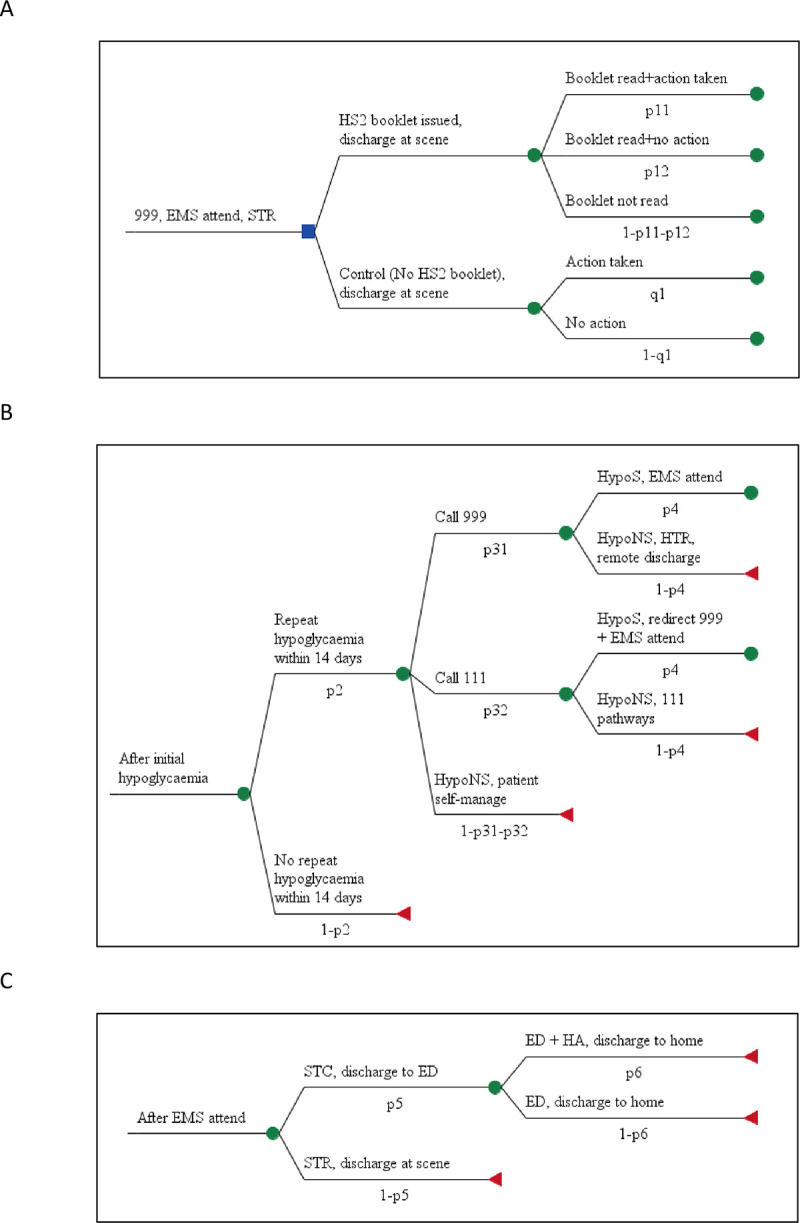
Decision tree structure of the economic model. (A) stage 1: responses to initial hypoglycaemia. (B) stage 2: responses to recurrent hypoglycaemia. (C) stage 3: secondary care outcomes. Full tree formed by mapping stage 3 onto every non-terminating node of stage 2, then that combination mapped onto every stage 1 branch.

The economic model is constructed as a decision tree in which outcomes involving recurrent hypoglycaemia are depicted for the target population over the fortnight following the initial attack. The model is displayed in Fig 1, with model states labelled and symbols given for the transition probabilities (values for which may be branch dependent).

For purposes of presentation the model is split into a sequence of 3 consecutive stages. The model’s first stage concerns patient options to respond arising from the initial hypoglycaemia, for example, on the extent to which the HS2 intervention is uptaken. The model’s second stage concerns patient and health services responses to recurrent hypoglycaemia. The model permits treatment to be sought from EMS (phoning 999), IUC (phoning 111) or to self-manage non-severe hypoglycaemia. It is assumed that the EMS and IUC despatch desks, respectively CAD (999 Computer aided despatch) and CAS (111 Clinical Assessment Service), correctly triage the incident to be either severe, HypoS, or non-severe, HypoNS. We assume that EMS clinicians only attend cases of severe hypoglycaemia. The model’s third stage concerns secondary care outcomes should the patient be conveyed to ED.

The full tree is built by mapping Stage 3 onto every non-terminating (green) node ending Stage 2 creating a combination that is then mapped onto every Stage 1 branch. In total there are 50 pathways in the model, 30 of which pertain to the HS2 intervention and 20 to standard care.

### Model costs

Table 2 lists the costs associated with each state of the economic model.

**Table 2:** Model costs.

State	Assigned cost (£)	Notes
***Stage 1***		
HS2 booklet issued	9.95	Intervention cost: HS2 booklet + HS2 administration (HS2 trial median duration 3.3 mins)
Control (no HS2 booklet)	0.00	
Booklet read+action taken	39.00	GP consultation
Booklet read+no action	0.00	
Action taken	39.00	GP consultation
No action	0.00	
***Stage 2***		
Repeat hypo within 14 days	0.00	
No repeat hypo within 14 days	0.00	
Call 999	7.33	999
Call 111	7.33	111
HypoNS, patient self-manage	(i) 25.03 (ii) 33.54	prop1*ED1NA+prop2*ED2NA+prop3*ED3NA+prop4*ED4NA+prop5*0 (self care): If HS2 booklet read+action taken: prop1 = prop2 = 0.08125, prop3 = prop4 = 0.01875, prop5 = 0.8 otherwise: prop1 = prop2 = 0.10625, prop3 = prop4 = 0.03125, prop5 = 0.725 Source: EPCG
HypoS, EMS attend	0.00	
HypoNS, HTR, remote discharge	47.49	HypoNS resolved by EMS HTR without need for onward referral for further NHS care
HypoS, redirect 999 + EMS attend	7.33	Additional call 999 due to IUC 111 pathway
HypoNS, 111 pathways	71.94	Weighted average of relative dispositions to NHS Pathways of call triaged by 111 CAS for 2018–19 (for ED cost sums ED(1,2)NA, for primary care ED(3,4)NA, for other at no cost). Source: NHS 111 Minimum Data Set 2018–19 [13] See file “20181009-N111WSI2-Weekly-NHS111-Collection-spec.pdf” paragraph “Dispositions”
***Stage 3***		
STC, discharge to ED	257.34	STC
STR, discharged at scene	209.38	STR
ED+HA, discharge to home	1478.13	HA plus weighted average of combined attendances ED1A and ED2A
ED, discharge to home	168.89	Weighted average of combined attendances ED1NA and ED2NA

The HS2 intervention is estimated to cost on average £9.95 per patient, where its administration time averages 3.3 minutes. (In the HS2 trial, the observed difference in median at-scene durations, HS2 versus standard care, was 6.6 mins for episodes occurring during the 8am-8pm period and 2 mins for episodes occurring overnight 8pm-8am. In the HS2 arm, 28% of episodes occurred 8am-8pm resulting in a weighted median HS2 administration time of 3.3 mins.) We assume that calls to NHS 111 are reimbursed equivalent to calls to 999. If hypoglycaemia recurs but is not severe (ie HypoNS), costs depend on whether the patient contacts EMS, IUC or self-manages the incident. Self-management for HypoNS is predominantly self-care at no cost to the NHS, but it also may involve self-transport to ED upon which NHS costs are incurred; the EPCG informed the breakdown by treatment arm.

### Transition probabilities

Table 3 lists the probabilities of transitioning between model states.

**Table 3:** Model transition probabilities.

Transition	HS2		Standard care		Notes
	symbol	Baseline probability	symbol	Baseline probability	
***Stage 1***					
Booklet read+action taken	p11	0.4125			Source: EPCG
Booklet read+no action	p12	0.3375			Source: EPCG
Action taken			q1	0.25	Source: EPCG
***Stage 2***					
Recurrent hypoglycaemia	p2	0.53 (read+action)	q2	0.53 (action taken)	Sources: Awalfi et al. [14] and EPCG
		0.59 (read only)		0.63 (no action)	
		0.63 (no actions)			
Call 999	p31	0.3625 (read+action)	q31	0.4 (action taken)	Source: EPCG
		0.4 (read only)		0.4 (no action)	
		0.4 (no actions)			
Call 111	p32	0.2375 (read+action)	q32	0.35 (action taken)	Source: EPCG
		0.35 (read only)		0.35 (no action)	
		0.35 (no actions)			
HypoS, EMS attend	p4	0.113	q4	0.121	Derived parameter, source HS2 trial
***Stage 3***					
STC, discharge to ED	p5	0.6	q5	0.6	Source: HS2 trial
ED+HA, discharge to home	p6	0.3333	q6	0.3333	Source: Esteves et al. [15]

Alwafi et al. [14] provide an incident rate range for hypoglycaemia of 0.072 to 42,890 episodes per 1,000 person-years, scaling to 2.8x10^-6^ to 1.65 episodes per person per fortnight. Assuming time to incident is exponentially distributed then the probability of hypoglycaemia is given by 1−*e*^−*r*^, where incident rate *r*<1.65 (= 42890x10^-3^/26). For recurrent hypoglycaemia, we set a trio of incident rates denoted (*r*_*NA*_, *r*_*HS*2*e*_, *r*_*HS*2_) that for deterministic analyses are assumed to satisfy the inequalities *r*_*HS*2_<*r*_*HS*2*e*_<*r*_*NA*_<1.65. Rate *r*_*NA*_ is due to no actions taken, *r*_*HS*2*e*_ and *r*_*HS*2_ are due to partial and full success of the HS2 intervention, respectively. After consultation with members of the study EPCG group, from an assumed mid-range value *r*_*NA*_ = 1, set were *r*_*HS*2_ = 0.75 and *r*_*HS*2*e*_ = 0.9. For probabilistic analyses (10,000 simulations) incident rates were assumed independently triangular distributed on support (0,1.65) with modes 0.75, 0.9 and 1 as above.

Repeat EMS attendance parameters, namely the transition probabilities *p*_4_ and *q*_4_, were tethered to the observed repeat attendance rates (intervention 4.5%, standard care 5.5%) being the primary outcome by arm measured in the HS2 trial. In the intervention arm of the model there are 18 (of 30) pathways in which EMS attendance occurs (12 of 20 for standard care). With both transition probabilities assumed constant in each pathway, their baseline values were derived as per: intervention ${\hat{p}}_{4}=0.045/\Sigma_{i}P_{i}=0.113$ and standard care ${\hat{q}}_{4}=0.055/\Sigma_{i}P_{i}=0.121$, where both sums range over every pathway contribution *P*_*i*_ by arm in which EMS attendance occurs. For probabilistic analyses, values for the pair *p*_4_ and *q*_4_ were this time matched to simulated repeat attendance rates, the latter generated from independent Binomial distributions (BN) with parameters set to values observed in the HS2 trial: intervention BN(707,4.5%) and standard care BN(1674,5.5%).

### Utilities

As a function of time *t*, let *U*(*t*) denote the patient health-related utility pathway, defined such that 0≤*U*(*t*)≤1 and which is monotonic in health improvement from states *U* = 0 (death) to *U* = 1 (perfect health). Over the fortnight (ie for 0<*t*<2) following the initial hypoglycaemia (at *t* = 0) assume constant *U*(*t*) = *U*_0_ if recurrent hypoglycaemia does not occur, where *U*_0_ = *U*(0). On the other hand, if hypoglycaemia recurs at time *t* = *T*<2 set *U*(*t*) = *U*_0_ for *t*<*T*, and *U*(*t*) = *U*_0_−Δ for *t*≥*T* for a societal utility decrement Δ>0 with values that depend on severity and time of day of incident: 0.004 for a daytime HypoNS; 0.007 for a nocturnal HypoNS; 0.062 for a daytime HypoS; 0.057 for a nocturnal HypoS [10]. (Beaudet et al. [16] propose the utility decrements (0.014,0.047) for (HypoNS, HypoS) that were reported by Currie et al. [17]) Note that the pathway assumed for recurring hypoglycaemia does not permit recovery back to the initial utility level within the two-week focus window.

Denote the per fortnight probability distribution of recurrent hypoglycaemia (none, HypoNS, HypoS) by (*p*_0_, *p*_*NS*_, *p*_*S*_), where 0<*p*_0_, *p*_*NS*_, *p*_*S*_<1 and *p*_0_+*p*_*NS*_+*p*_*S*_ = 1, and utility decrements associated with (HypoNS, HypoS) by (Δ_*NS*_, Δ_*S*_) = 0.006,0.058), where these have been averaged by the proportion of daytime incidents, 28%, and nocturnal incidents, 72%, that were observed in the HS2 trial. Using superscripts SC and HS2 to indicate standard care and the HS2 intervention, respectively, the difference of the annualised area-under-the-curve for each utility pathway is

$$\frac{9}{182}\left(0.006(p_{NS}^{SC}-p_{NS}^{HS2})+0.058(p_{S}^{SC}-p_{S}^{HS2})\right)$$ (1)

where this has been subject to averaging with respect to the distribution of *T*, assumed to be triangular on support (0,2) with mode at 2 days. This gives the denominator in the incremental cost-effectiveness ratio (ICER) that expresses added cost per additional QALY due to the HS2 intervention when compared to standard care.

### Cost minimisation model

**Fig 2:**
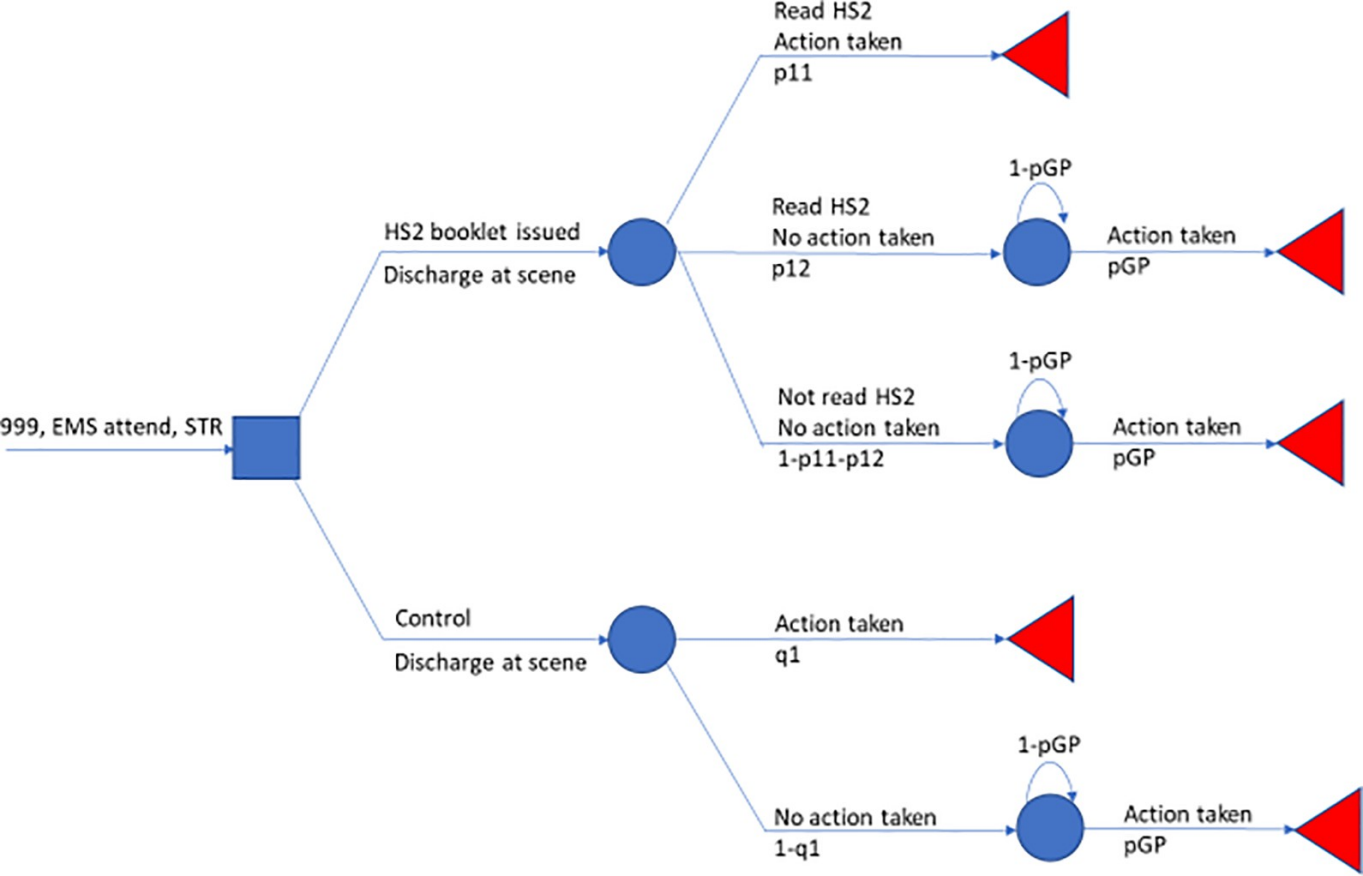
Cost minimisation model. Model is depicted by patient types in the HS2 intervention arm and in the standard care arm. Once the first fortnight-length cycle concludes exposure to risk of recurrent hypoglycaemia in each subsequent cycle replicates until GP attendance, where the latter defines the absorbing event for the Markov chains.

For the purposes of conducting a cost minimisation analysis we add simple, independent absorbing markov chains of fortnight-length cycle for patient type to a modified version of the economic model, terming this the cost minimisation model. In the first cycle, Stage 1 of the economic model is modified such that “booklet read+action taken” in the HS2 arm and “action taken” in the standard care arm become absorbing states. The remaining patient types—in the HS2 arm “booklet read but no action taken” and “no actions taken”, in the standard care arm “no action taken”–each have as absorbing state “action taken” for which a common absorption transition probability is assumed, values applied (0.3,0.4,0.5). The cost minimisation model is displayed in Fig 2.

Costs for the continuation states correspond to those generated in the first cycle for each patient type. The absorption state attracts a once-only cost, GP. Total costs per cycle are accumulated, and the particular cycle at which the accumulation under the HS2 intervention is strictly less than that of standard care is recorded. Recording is not triggered if absorption in both arms has exceeded 95% which, when that first occurs, is the iterating stopping rule. The probabilistic settings as previously described are used for the first cycle.

### Computations

All computations were undertaken using Mathematica® version 13.2 (free player available at https://www.wolfram.com/player/) and mathStatica® version 2.72. Commands appear in “Computations.nb” uploaded to S3 File.

## Results

### Efficacy

The HS2 trial’s primary outcome, recurrent hypoglycaemia within a fortnight of the initial attack, was measured in the intervention arm as 32 repeats in 707 STR episodes (4.5%). In the standard care arm the corresponding count was 92 repeats in 1674 STR episodes (5.5%). For the two-sided test of equality in proportions (two-proportion z-test), the probability value in support of the equality hypothesis was p = 0.33. There is no statistically significant difference in recurrent hypoglycaemia within a fortnight of the initial attack when the HS2 intervention was implemented versus standard care alone.

### Baseline

NHS cost incurred per patient receiving the HS2 intervention over the fortnight following an initial hypoglycaemia are predicted to average £49.79. Almost one-half of that total, £24.60 (49.4%), which includes the intervention cost £9.95, is due to EMS. The remaining £25.19 of average NHS cost is distributed across primary care, IUC and secondary care service providers. Under standard care, per patient NHS costs average £40.50, of which £16.56 (40.9%) is due to EMS.

Representing patient benefit by the probability of no recurrence of hypoglycaemia, the model predicts this to be on average 42.4% for the patient subject to the HS2 intervention and 39.4% under standard care. This amounts to a 7.6% reduction in relative risk of recurrent hypoglycaemia due to implementation of the HS2 intervention versus standard care.

Upscaling units to whole numbers of recurrent hypoglycaemia, the modelled estimate of the ICER for the HS2 intervention versus standard care is 309.36 (= 100(49.79–40.50)/(42.4–39.4)). To the assumptions given and a time horizon of one fortnight beyond the initial hypoglycaemia implementing the HS2 intervention will cost the NHS an additional £309.36 per episode of recurrent hypoglycaemia avoided. Baseline results are summarised in Table 4.

**Table 4:** Baseline modelling results.

Service provider	Average per patient cost by service provider (£)		Cost difference (£)	Per patient probability of recurrent hypoglycaemia avoided		Probability difference	ICER (£/recurrent hypoglycaemia avoided)
	HS2	Standard Care		HS2	Standard Care		
EMS	24.60	16.56	8.04				
IUC	0.41	0.54	-0.13				
Primary care	13.27	10.19	3.07				
Secondary care	11.51	13.20	-1.69				
TOTAL	49.79	40.50	9.29	0.424	0.394	0.030	309.36

Finally, a further way to view cost is by patient type according to individual response to the HS2 intervention. Model estimates of the cost the NHS incurs to manage recurrent hypoglycaemia in the patient: (i) who read the HS2 booklet and took action averages £75.71; (ii) who only read the HS2 booklet averages £46.66; and (iii) who took no action whatsoever averages £49.05. Estimates under standard care: (i) of the patient who took action by consulting their GP or diabetic nurse costs the NHS £72.14 on average; and (ii) of the patient who took no action costs the NHS on average £39.70.

### Sensitivity analyses

#### Intervention cost

The time taken for EMS clinicians to organise an appointment for the patient to consult their GP contributes the greater part of the intervention cost. Should the incident occur outside of business hours, as occurred in 72% of episodes in the HS2 trial, then the consultation appointment cannot be organised. In this event, the added time for episodes occurring out-of-hours averaged 2 minutes, which we attribute wholly as the referral duration due to HS2. If confined to conduct outside of business hours the HS2 intervention cost falls from £9.95 to £6.70 and its ICER falls to £201.14 per hypoglycaemia avoided, all other factors held constant. Alternately, when the HS2 intervention is conducted during business hours the time added to episode length averaged 6.6 minutes in the HS2 trial. If confined to conduct during business hours the HS2 intervention cost increases to £18.43 and the ICER to £591.66 per hypoglycaemia avoided, all other factors held constant.

#### Recurrence of hypoglycaemia

The hypoglycaemia repeat rate depends on patient actions such as whether their GP or diabetic nurse is consulted, and, if so, the follow-on improvements arising from revisions in their medicine management. If improvements in medicine management are more substantive than anticipated, then the ICER will fall. For example, if baseline *r*_*HS*2_ is halved to 0.375, then the ICER falls to £128.77 per hypoglycaemia avoided, all other factors held constant.

#### Severe hypoglycaemia

All episodes of severe recurrent hypoglycaemia (HypoS) are, by model assumption, attended at-scene by EMS clinicians. Should HypoS for those receiving the HS2 intervention increase from baseline 4.5% to 5.5%, matching the rate observed under standard care in the HS2 trial, the ICER increases to £363.76 per hypoglycaemia avoided, all other factors held constant. On the other hand, should it drop to 3.5%, at which point the p-value for the HS2 trial primary outcome drops below 0.05, the ICER decreases to £254.82 per hypoglycaemia avoided, all other factors held constant.

**Fig 3:**
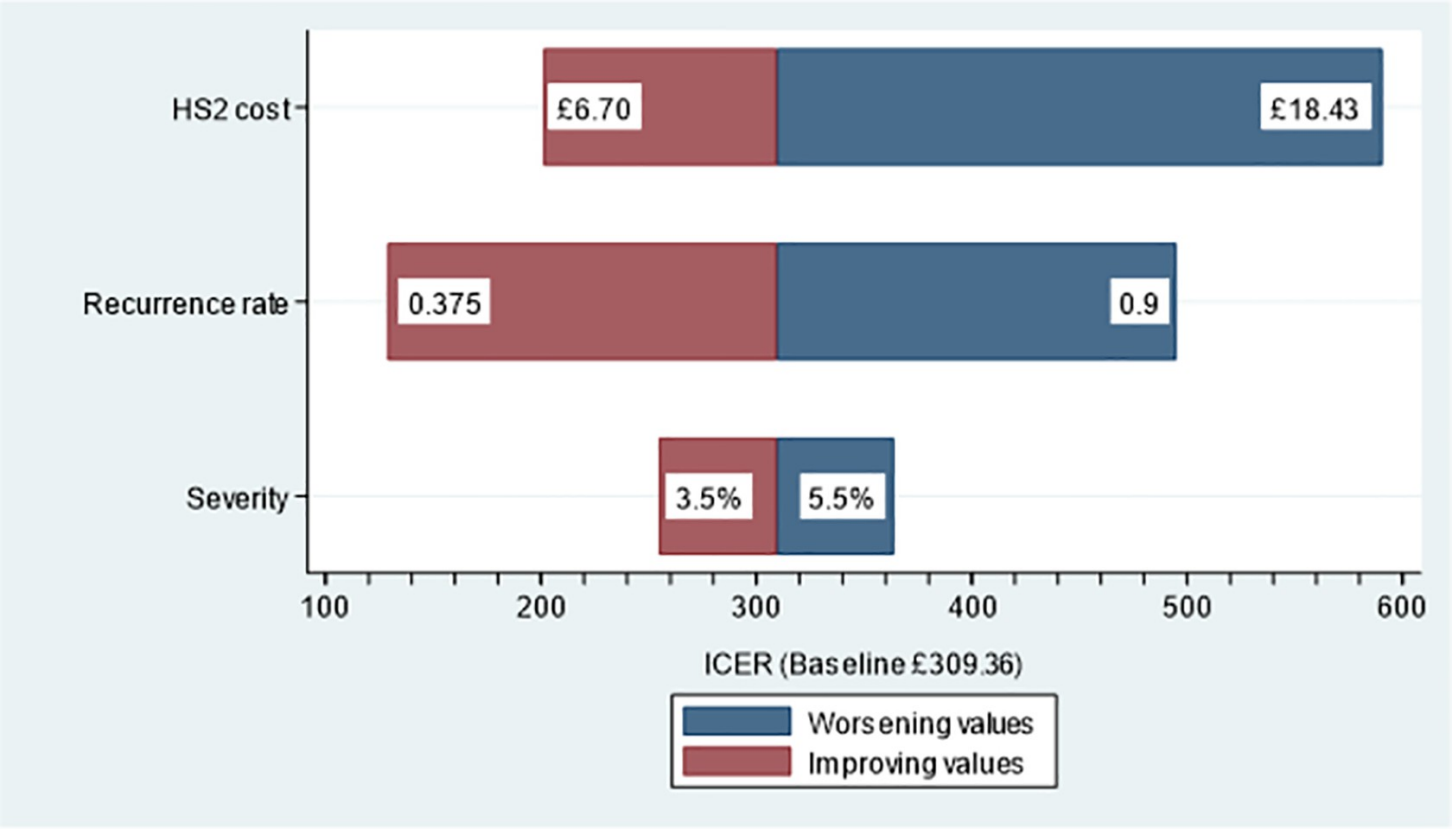
Tornado diagram for selected sensitivity analyses. One-way sensitivities of baseline ICER due to low/high variations: (i) HS2 intervention cost (+£6.70 to +£18.43; baseline £9.95), (ii) Hypoglycaemia recurrence rate *r*_*HS*2_ (0.375 to 0.9; baseline 0.75), (iii) Severe recurrent hypoglycaemia rate (3.5% to 5.5%; baseline 4.5%).

A tornado diagram depicting the results of the three sensitivity analyses is displayed in Fig 3.

### Probability analysis

**Fig 4:**
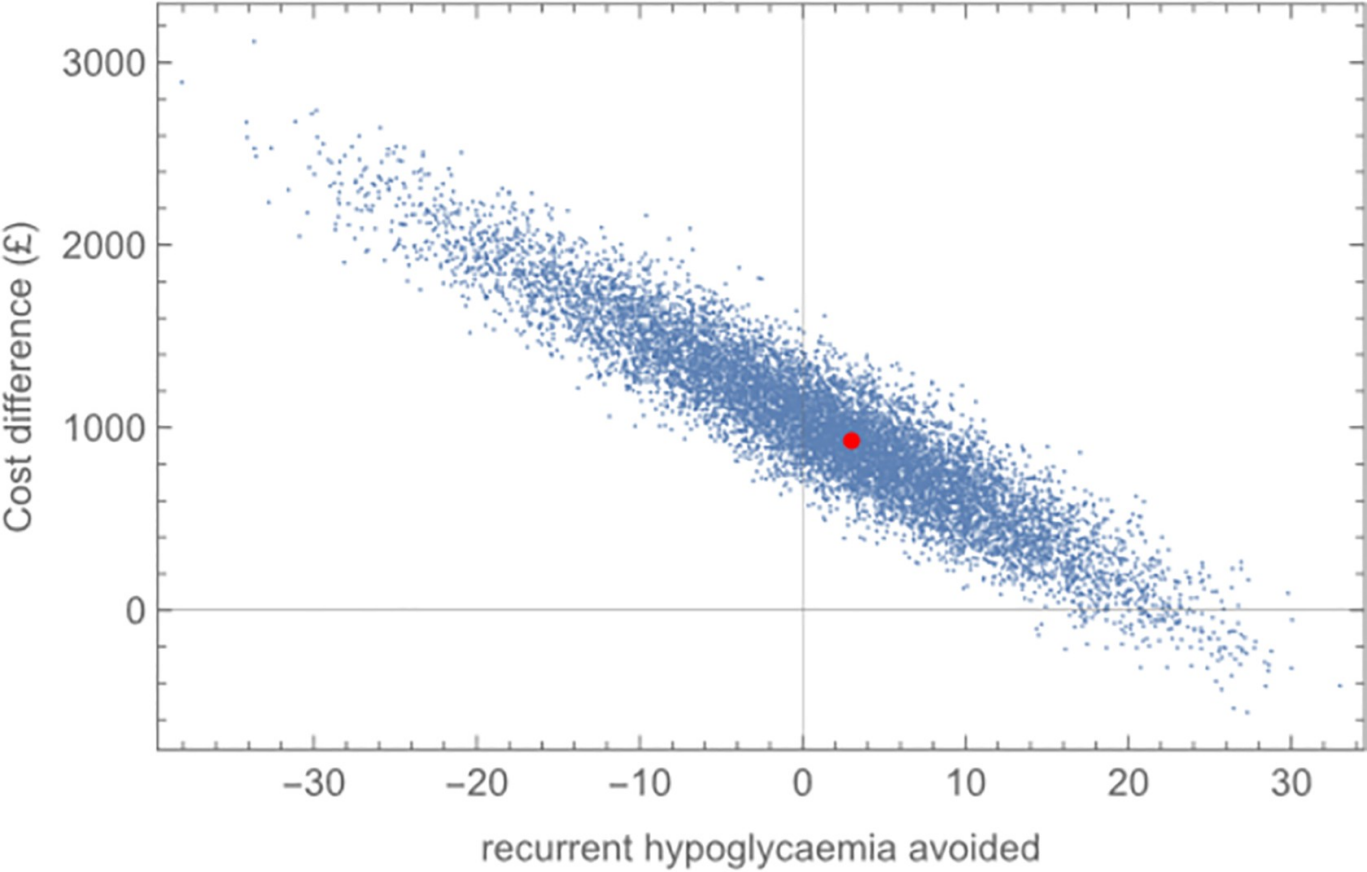
Cost-effectiveness plane (baseline value shown in red). Cost differential between care under HS2 intervention and standard care plotted against number of recurrent hypoglycaemia cases avoided. 10,000 simulated pairs in which recurrent hypoglycaemia incidence and severity rate vary. Baseline (red) cost difference +£929 for +3.00 avoided cases.

Parameters varied in the probabilistic analysis of the baseline model were incidence rates of recurrent hypoglycaemia for each patient type as well as rates of severe HypoS cases. All other parameters, apart from the derived transition probabilities *p*_4_ and *q*_4_ which update in each simulation, were held at their baseline settings. In total, 10,000 simulations were performed. The cost-effectiveness plane, in which the simulated cost differential between the HS2 intervention and standard care is plotted against the simulated number of recurrent hypoglycaemia cases avoided, is shown in Fig 4.

The positioning of the cloud of points is such that we identify a significant chance that the HS2 intervention is dominated by standard care (ie the HS2 intervention is both costlier and less effective than standard care), there being 41.81% of all realisations falling in the upper left quadrant of the cost-effectiveness plane. In contrast, the proportion of realisations falling in the lower right quadrant, being 1.28%, provides the estimate of the chance that the HS2 intervention will dominate standard care.

### Cost-utility

By severity, the baseline model estimates of the probability distribution of recurrent hypoglycaemia (none, HypoNS, HypoS) are:

undertheHS2intervention(42.4%,53.1%,4.5%)

understandardcare(39.4%,55.1%,5.5%)

Substitution into (1) and dividing the baseline per person cost difference, £49.79–40.50 = £9.29, by it yields ICER = 270,000 (£/QALY). This value is well beyond any commissionable threshold in the NHS. To the assumptions given and a time horizon of one fortnight beyond the initial hypoglycaemia the HS2 intervention is not cost-effective against standard care in its use of NHS resources to reduce the risk of recurrent hypoglycaemia.

### Cost minimisation

**Fig 5:**
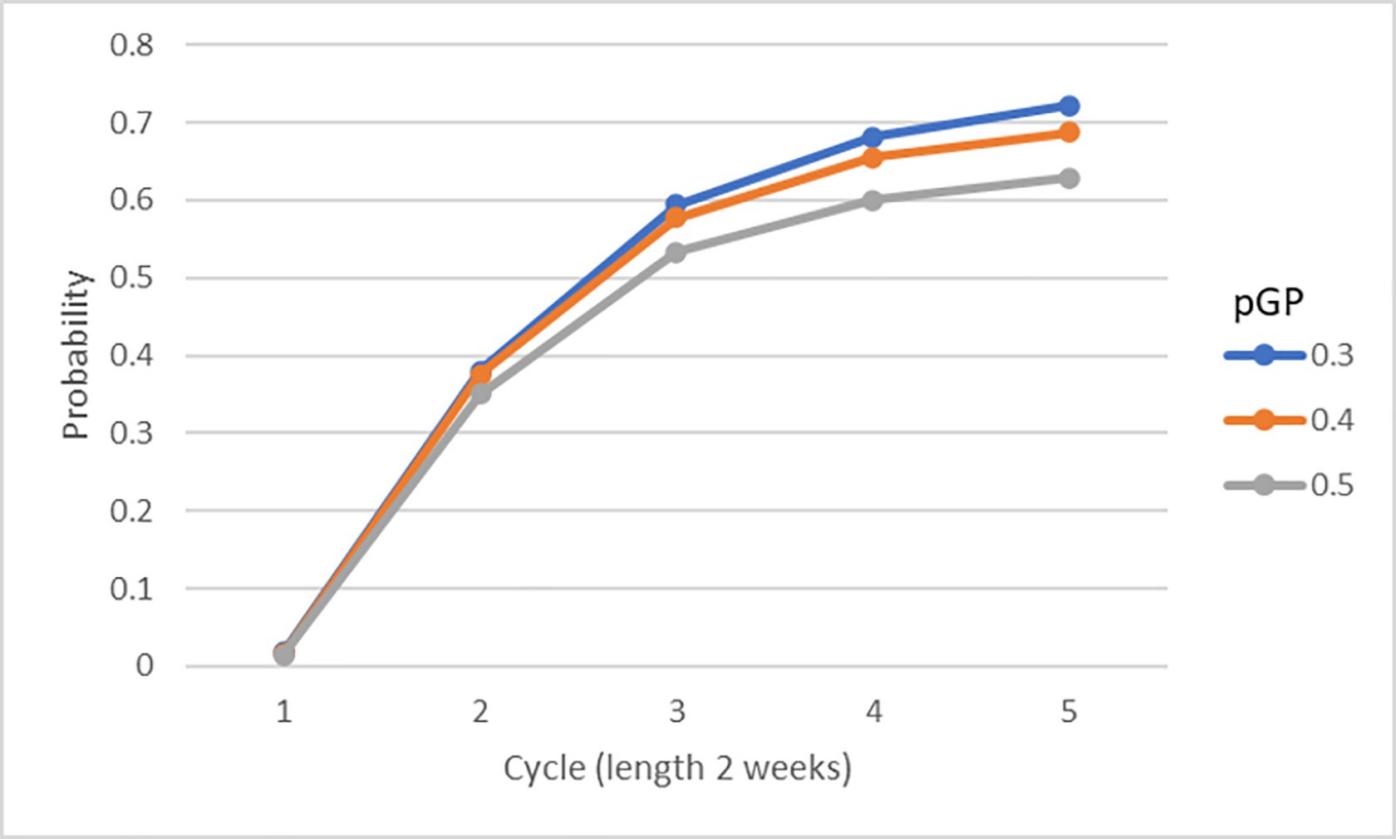
Probability HS2 is least total cost versus standard care by completed cycle, for GP consultation rates 0.3, 0.4 and 0.5. Estimates of probability that care under HS2 intervention has lesser cost than standard care after n = 1,2,3,4,5 fortnight-length cycles, by absorption probability pGP = 0.3,0.4,0.5. Estimates depicted are proportion of 10,000 simulations in which first-cycle recurrent hypoglycaemia incidence and severity rate vary, then replicate in up to n-1 subsequent cycles until HS2 is least cost.

For a given value of the absorption probability (ie GP consultation rate), 10,000 simulations of the cost minimisation model were run from which were formed the count of cases such that total costs of the HS2 intervention were less than those of standard care at the conclusion of a given number of cycles. Expressed as proportions, these are plotted in Fig 5 for model cycles 1 through 5 by the values set for the absorption probability.

Generally, the higher is the GP consultation rate assumed from cycle 2 the lesser is the difference between the HS2 intervention and standard care implying lesser chance that the former will minimise total cost at every cycle point. Averaging proportions across the consultation rates (pGP = 0.3,0.4,0.5), after, for example, 2 complete cycles (ie 4 weeks beyond the initial incident) the probability that the HS2 intervention will have lesser total cost than standard care averages 37%. After 3 / 4 / 5 cycles (ie 6 / 8 / 10 weeks) the average increases to 57% / 65% / 68%.

## Discussion

We constructed a de novo economic model to evaluate across differing paradigms, deterministic and probabilistic, the ambulance clinician HS2 intervention designed to reduce the risk of recurrent hypoglycaemia for a period up to two weeks beyond an initial attack. Cost-effectiveness analysis revealed the cost to the NHS per case of recurrent hypoglycaemia avoided exceeded £300. Using societal utility weights for severity of recurrent hypoglycaemia, cost-utility analysis revealed that the HS2 intervention was not cost-effective against standard NHS care. The lack of economic evidence found to support introduction of the HS2 intervention in its current form mirrors a similar lack of statistical modelling support on two weeks outcomes for HS2 reported by Botan et al. [5; supplementary material and results, table S.A1].

Prior evidence is ambivalent in its support for leaflet-based interventions. For example, O’Cathain et al. [18] report that evidence-based leaflets were ineffective in promoting informed choice in women using maternity services, Nitschke et al. [19] indicated only potential usefulness of leaflets to enable older people to remain healthy during periods of extreme heat, whereas Sankhar et al. [20] found evidence in support of an educational leaflet provided it was targeted to literate hypoglycaemic patients in India. Mason et al. [21] report significant support for a complex intervention involving an information leaflet versus an information leaflet alone, although much earlier evidence from Eaden et al. [22] opposed this. The HS2 intervention has features of both depending on time of day as to whether it is possible to arrange a next-day appointment for the patient with their GP.

HS2 is a low-cost intervention estimated to average £9.95 per treated patient, this is significantly more than the estimate of £3.70 given in Botan et al. [5]. The major cost component attributable to the HS2 intervention is due to its implementation time. Filling out the HS2 booklet occupied EMS clinicians on average approximately 2 minutes. Additional time, averaging over 4 minutes, arose during business hours when the clinician organises a next-day appointment for the patient to consult their GP about their diabetic management. Outside of business hours, when that appointment cannot be made, intervention cost falls as does the ICER. The model did not attempt to account for parameter variation in regard to time of day of the initial incident, nor indeed to allow for nonadherence in attending the consultation.

Cost minimisation assumed that the difference in benefit between the HS2 intervention and standard care is temporary, vanishing to zero when a patient consults their GP or diabetic nurse as part of routine diabetes care. This is consistent with the findings of Doi-Kanno et al. [23] in which a leaflet-based intervention had only short-term effect. In terms of cost, while Botan et al. [5] claim HS2 saves on cost to the NHS over standard care, we in contrast estimate the chance of that event to be negligible when assessed against the two week primary outcome, and no more than 70% for a secondary outcomes horizon extended up to ten weeks beyond the initial attack.

## Conclusion

The HS2 intervention is not a cost-effective use of NHS resources when compared to standard NHS care in reducing the risk of hypoglycaemia recurring within a fortnight of an initial attack that was resolved at-scene by EMS ambulance clinicians.

## Supporting information

S1 File Abbreviations and acronyms. (DOCX)

S2 File Ambu-HS2 protocol v1.1. (DOCX)

S3 File Computations. (NB)

## References

[1] Barendse S, Singh H, Frier BM, Speight J. The impact of hypoglycaemia on quality of life and related patient-reported outcomes in Type 2 diabetes: a narrative review. Diabet Med 2012;29:293–302. doi: 10.1111/j.1464-5491.2011.03416.x 21838763

[2] Seaquist ER, Anderson J, Childs B, Cryer P, Dagogo-Jack S, Fish L, et al. Hypoglycemia and diabetes: a report of a workgroup of the American Diabetes Association and the Endocrine Society. Diabetes Care 2013;36:1384–95. doi: 10.2337/dc12-2480 23589542 PMC3631867

[3] Heller SR, Frier BM, Herslov ML, Gundgaard J, Gough SCL. Severe hypoglycaemia in adults with insulin-treated diabetes: impact on healthcare resources. Diabet Med 2016;33:471–7. doi: 10.1111/dme.12844 26179360 PMC5034744

[4] Fitzpatrick D, Duncan EAS. Improving post-hypoglycaemic patient safety in the prehospital environment: a systematic review. Emerg Med J 2009;26:472–8. doi: 10.1136/emj.2008.062240 19546265

[5] Botan V, Law GR, Laparidou D, Rowan E, Smith MD, Ridyard C, et al. The effects of a leaflet-based intervention, ‘Hypos can strike twice’, on recurrent hypoglycaemic attendances by ambulance services: A non-randomised stepped wedge study. Diabet Med 2021 Oct;38(10):e14612. doi: 10.1111/dme.14612 34053095

[6] Laparidou D, Botan V, Law G, Rowan E, Smith MD, Brewster A, et al. People with diabetes and ambulance staff perceptions of a booklet-based intervention for diabetic hypoglycaemia, “Hypos can strike twice”: a mixed methods process evaluation. BMC Emerg Med 2022 22:21. doi: 10.1186/s12873-022-00583-y 35135499 PMC8822761

[7] ClinicalTrials.gov [Internet]. Bethesda (MD): National Library of Medicine (US). 2020 January 28—Identifier NCT04243200, Ambulance ’Hypos Can Strike Twice’ Study (Ambu-HS2). Available from: https://clinicaltrials.gov/ct2/show/NCT04243200

[8] Khunti K, Fisher H, Paul S, Iqbal M, Davies MJ, Siriwardena AN. Severe hypoglycaemia requiring emergency medical assistance by ambulance services in the East Midlands: a retrospective study. Prim Care Diabetes 2013;7:159–65. doi: 10.1016/j.pcd.2013.01.001 23375384

[9] Department of Health and Social Care [Internet]. National Schedule of NHS Costs 2018–19. Available at https://www.england.nhs.uk/publication/2018-19-national-cost-collection-data-publication/ (accessed December 2021)

[10] Evans M, Khunti K, Mamdani M, Galbo-Jorgensen CB, Gundgaard J, Bogelund M, et al. Health-related quality of life associated with daytime and nocturnal hypoglycaemic events: a time trade-off survey in five countries. Health Qual Life Outcomes 2013;11:90. doi: 10.1186/1477-7525-11-90 23731777 PMC3679729

[11] Curtis L, Burns A. Unit Costs of Health and Social Care 2019. Unit Costs of Health and Social Care. PSSRU, Kent, UK, 176 pp. ISBN 978-1-911353-10-2

[12] Siriwardena AN, Law G, Smith MD, Iqbal M, Phung V-H, Spaight A, et al. Predictors and outcomes of ambulance calls to diabetes-related emergencies in care homes–retrospective observational database study. Report to NIHR CLAHRC East Midlands, 2018.

[13] NHS 111 Minimum Data Set 2018–19. Available at https://www.england.nhs.uk/statistics/statistical-work-areas/nhs-111-minimum-data-set/ (accessed March 2021)

[14] Awalfi H, Alsharif AA, Wei L, Langan D, Naser AY, Mongkhon P, et al. Incidence and prevalence of hypoglycaemia in type 1 and type 2 diabetes individuals: A systematic review and meta-analysis. Diabetes Res Clin Pract 2020;170:108522. doi: 10.1016/j.diabres.2020.108522 33096187

[15] Esteves C, Neves C, Sá JJ, Carvalho D. Severe hypoglycaemia in diabetic patients in Pre‑hospital and Emergency Department care: a cross‑sectional survey. BMC Res Notes 2018;11:249. doi: 10.1186/s13104-018-3363-0 29685177 PMC5914041

[16] Beaudet A, Clegg J, Thuresson P-O, Lloyd A, McEwan P. Review of utility values for economic modeling in type 2 diabetes. Value Health 2014;17:462–70. doi: 10.1016/j.jval.2014.03.003 24969008

[17] Currie C, Morgan C, Poole C, Sharplin P, Lammert M, McEwan P. Multivariate models of health-related utility and the fear of hypoglycaemia in people with diabetes. Curr Med Res Opin 2006;22:1523–34. doi: 10.1185/030079906X115757 16870077

[18] O’Cathain A, Walters S, Nicholl J, Thomas K, Kirkham M. Use of evidence based leaflets to promote informed choice in maternity care: randomised controlled trial in everyday practice. BMJ 2002;324:1–5. doi: 10.1136/bmj.324.7338.643 11895822 PMC84396

[19] Nitschke M, Krackowizer A, Hansen A, Bi P, Tucker G. Heat health messages: A randomized controlled trial of a preventative messages tool in the older population of South Australia. Int J Environ Res Public Health 2017;14:992. doi: 10.3390/ijerph14090992 28858262 PMC5615529

[20] Sankhar V, Sherif A, Sunny A, John G, Rajasekaran S. The impact of patient information leaflets to prevent hypoglycemia in out-patients with type 2 diabetes mellitus. Ars Pharm 2019;60:5–14. doi: 10.30827/ars.v60i1.7614

[21] Mason F, Farley A, Pallan M, Sitch A, Easter C, Daley A. Effectiveness of a brief behavioural intervention to prevent weight gain over the Christmas holiday period: randomised controlled trial. BMJ 2018;363:k4867. doi: 10.1136/bmj.k4867 30530821 PMC6287121

[22] Eaden J, Abrams K, Shears J, Mayberry J. Randomized controlled trial comparing the efficacy of a video and information leaflet versus information leaflet alone on patient knowledge about surveillance and cancer risk in ulcerative colitis. Inflamm Bowel Dis 2002;8:407–12. doi: 10.1097/00054725-200211000-00005 12454616

[23] Doi-Kanno M, Kanoya Y, Moriguchi E. The effects of a leaflet-based health guide on health literacy, self-efficacy, and satisfaction among older Japanese-Brazilian adults living in Brazil: A quasi-experimental study. BMC Public Health 2021;21:10. doi: 10.1186/s12889-020-10129-1 33397356 PMC7784267

