# Supplementary material for: Model-based economic evaluation of the effectiveness of “‘Hypos’ can strike twice”, a leaflet-based ambulance clinician referral intervention to prevent recurrent hypoglycaemia
Source: PLoS One. 2023 Mar 16;18(3):e0282987. doi: 10.1371/journal.pone.0282987 (PMC10019663; doi:10.1371/journal.pone.0282987)
Supplement: S1 File — (DOCX) [file pone.0282987.s001.docx]

**Selected list of abbreviations and acronyms**

CAD Emergency service call NHS 999 computer aided despatch

CAS Urgent Care service call NHS 111 clinical assessment service

ED Emergency Department

EDxA Emergency medicine attendance at Type x accident and emergency department,

patient admitted into hospital care (x=1,2,3,4)

EDxNA Emergency medicine attendance at Type x accident and emergency department,

patient discharged (x=1,2,3,4)

EMS Emergency Medical Services

EPCG Expert Patient and Clinician Group

GP General practitioner, family doctor

HA Hospital admission

HS2 ‘Hypos’ can strike twice

HTR Ambulance service hear, treat and/or refer

HypoNS non-severe (mild/moderate) recurrent hypoglycaemic attack

‘Hypos’ Hypoglycaemic attack

HypoS severe recurrent hypoglycaemic attack

ICER incremental cost-effectiveness ratio

IUC Integrated Urgent Care

NHS UK National Health Service

QALY Quality adjusted life-year

STC Ambulance service see, treat and convey

STR Ambulance service see, treat and/or refer

UK United Kingdom
