# Supplementary material for: Model-based economic evaluation of the effectiveness of “‘Hypos’ can strike twice”, a leaflet-based ambulance clinician referral intervention to prevent recurrent hypoglycaemia
Source: PLoS One. 2023 Mar 16;18(3):e0282987. doi: 10.1371/journal.pone.0282987 (PMC10019663; doi:10.1371/journal.pone.0282987)
Supplement: S2 File — (DOCX) [file pone.0282987.s002.docx]

| **PROTOCOL**  Effect of implementing an ambulance clinician delivered hypoglycaemia intervention (‘Hypos can strike twice’) on repeat ambulance calls, attendances and transportation to hospital: non-randomised stepped wedge and process evaluation  Ambulance ‘Hypos can Strike Twice’ study  **Ambu-HS2**  Protocol Version  ***1.1***  Date 03.03.2020 | |
| --- | --- |
| IRAS Project ID | 276 438 |
| Registration ID | ClinicalTrials.gov Identifier: NCT04243200 |
| Sponsor | University of Lincoln |
| Sponsor ID | 191202 |
| Funder | NIHR Applied Research Collaboration (ARC) East Midlands |


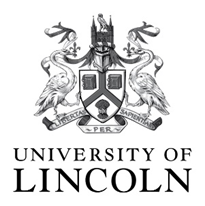


SIGNATURE PAGE

The undersigned confirm that the following protocol has been agreed and accepted and that the Chief Investigator agrees to conduct the study in compliance with the approved protocol and will adhere to the principles outlined in the Declaration of Helsinki, the Sponsor’s SOPs, and other regulatory requirement(s).

I agree to ensure that the confidential information contained in this document will not be used for any other purpose other than the evaluation or conduct of the investigation without the prior written consent of the Sponsor

I also confirm that I will make the findings of the study publicly available through publication or other dissemination tools without any unnecessary delay and that an honest accurate and transparent account of the study will be given; and that any discrepancies from the study as planned in this protocol will be explained.

Chief Investigator:

Signature: ............................................................................. Date: ....../....../......

Name: Aloysius Niroshan Siriwardena

# STUDY/TRIAL CONTACTS

| Chief Investigator | Name: A. Niroshan Siriwardena  Address: University of Lincoln, Sarah Swift Building, Brayford Wharf East, Lincoln, LN5 7AY, UK  UoL job title: Professor of Primary and Prehospital Health Care  Phone: 01522 886939  Email: nsiriwardena@lincoln.ac.uk |
| --- | --- |
| Sponsor  Contact details: | University of Lincoln  Andrew Stevenson  Director Research and Enterprise  University of Lincoln  Bridge House  Brayford Pool  Lincoln  LN6 7TS |
| Collaborators/Co-Investigators/Protocol Contributors | Name: Mrs Amanda Brewster  Job title: Public and Patient Involvement representative  Phone:  Email: abrewster747@btinternet.com |
|  | Name: Mr Keith Spurr  Job title: Public and Patient Involvement representative  Phone:  Email: keithspurr@msn.com |
|  | Name: Mrs Pauline Mountain  Job title: Public and Patient Involvement representative  Phone:  Email: gomoun@aol.com |
|  | Name: Mrs Sally Dunmore  Job title: Ambulance Implementation Lead  Phone:  Email: sally.dunmore@emas.nhs.uk |
|  | Name: Mrs June James  Job title: Diabetes Nurse Specialist  Phone:  Email: jj194@le.ac.uk |
|  | Name: Dr Murray D. Smith  Job title: Econometrician and Health Economist  Phone: 01522 886785  Email: mdsmith@lincoln.ac.uk |
|  | Name: Ms Despina Laparidou  Job title: Research assistant  Phone: 01522 837407  Email: dlaparidou@lincoln.ac.uk |
|  | Name: Dr Elise Rowan  Job title: Clinical Database Manager  Phone: 01522 886481  Email: erowan@lincoln.ac.uk |
|  | Name: Dr Leon Roberts  Job title: Ambulance Service Medical Director  Phone:  Email: Leon.Roberts@emas.nhs.uk |
|  | Name: Prof Kamlesh Khunti  Job title: Co-investigator  Phone:  Email: kk22@leicester.ac.uk |
| Statistician | Name: Prof Graham Law  Job title: Statistician  Phone: 01522 835762  Email: glaw@lincoln.ac.uk |

# FUNDER DETAILS

| **FUNDER(S)** | **FINANCIAL AND NON-FINANCIAL SUPPORT GIVEN** |
| --- | --- |
| NIHR Applied Research Collaboration (ARC) East Midlands | £ 65,618.17 |
| University of Lincoln | In-kind contributions from Professors Law (5% FTE), and Siriwardena (5% FTE). |
| University of Leicester | In-kind contributions from Professor Khunti (5% FTE). |
|  | There is an additional contribution of £1000 from Professor Khunti via his NIHR Senior Investigator Award. |

# STUDY SUMMARY

| Study Title | Effect of implementing an ambulance clinician delivered hypoglycaemia intervention (‘Hypos can strike twice’) on repeat ambulance calls, attendances and transportation to hospital: non-randomised stepped wedge and process evaluation |
| --- | --- |
| Study Design | Non-randomised stepped wedge design with mixed-methods process evaluation. |
| Study Participants | **Patients**  Young adults aged 16-17 years and adults aged 18 years+ with diabetes experiencing hypoglycaemia needing an ambulance service response.  **Clinicians**  Ambulance staff providing treatment and advice to people who have had a hypoglycaemic attack. |
| Eligibility Criteria | **Patients**  Young adults aged 16-17 years and adults aged 18 years+ with diabetes experiencing hypoglycaemia needing an ambulance service response. Hypoglycaemia for this study was identified as a ‘clinical impression’ of ‘hypoglycaemia’ or ‘diabetic problem’. Hypoglycaemia may also be identified according to ambulance guidelines^1^ as any blood glucose less than 4mmol/L.  **Clinicians**  Ambulance staff providing treatment and advice to people who have had a hypoglycaemic attack. |
| Planned Sample Size | Interviews: 10-15 staff and 10-15 patients, based on previous experience of the sample required to achieve theoretical saturation.  Survey: We will survey all front-line staff. The estimated sample size for patients is 447. |
| Study Duration | 12 months |
| Objectives | Aim: To evaluate the effect of implementing the ‘Hypo’s can strike twice’ intervention to patients with diabetes and hypoglycaemia attended by East Midlands Ambulance Service NHS Trust in the East Midlands region.  Objectives: To investigate the effect of implementing the ‘Hypo’s can strike twice’ on:   1. Repeat ambulance calls and attendances for hypoglycaemia; 2. Recorded referrals to an appropriate healthcare professional (e.g. GP, nurse); 3. Completed care bundle for hypoglycaemia (proportion of patients with all of blood glucose recorded before treatment, blood glucose recorded after treatment and treatment given for hypoglycaemia; 4. Costs of implementation ‘Hypo’s can strike twice’ intervention vs costs of health service resource use; 5. To conduct a process evaluation to explore how ‘Hypo’s can strike twice’ exerts its effects and can be scaled up if effective by understanding implementation, mechanisms of impact, and contextual factors using interviews and surveys. |
| Outcomes | Primary outcome   1. Repeat ambulance calls and attendances for hypoglycaemia within 14 days.   Secondary outcomes   1. Repeat ambulance calls and attendance for hypoglycaemia within 30 and 90 days. 2. Recorded referral to an appropriate healthcare professional (e.g. GP, nurse). 3. Completed care bundle for hypoglycaemia (proportion of patients with all of blood glucose recorded before treatment, blood glucose recorded after treatment and treatment given for hypoglycaemia. 4. Costs of implementation of ‘Hypo’s can strike twice’. 5. Process evaluation to explore how ‘Hypo’s can strike twice’ exerts its effects and can be scaled up if effective by understanding implementation, mechanisms of impact, and contextual factors using interviews and surveys |
| Data Analysis | Work package (WP) 1 Stepped wedge: Dispatch data will be linked with clinical data using a unique job number. People included will be identified and tracked for repeat attendances. In the stepped wedge design, the intervention and control observation periods take the place of the treatment and control groups in a parallel trial and the distribution of results across unexposed observation periods is compared with that across the exposed observation periods. There are a small number of steps and so characteristics of the groups in the wedge can be compared by group, including number, the average cluster size, cluster characteristics, and important patient characteristics.  Following an intention-to-treat principle, clusters will be analysed according to their crossover time irrespective of whether crossover was achieved at the desired time. Under the stepped wedge design the evaluation happens over a period of time, during which the number of clusters exposed to the intervention gradually increases. This means that unexposed observations will, on average, be from an earlier calendar time than exposed observations. Additionally, policy and service delivery changes may occur. Calendar time is associated with both the exposure to the intervention and also possibly the outcome, and so is a potential confounder and will be adjusted for in the analysis.  A linear mixed model will be fitted adjusting for the systematically different observation periods and for clustering in the data. Time is included as a fixed effect and a random effect for cluster. The design also allows examination of the impact of the intervention changing over time once it is introduced into a cluster.  WP2 Health economics: We will use a cost consequences model, estimating costs from NHS Reference Costs to evaluate the costs of the intervention in relation to its effects. Key cost drivers associated with repeat hypoglycaemic episodes will be modelled.  WP3 Survey: We will analyse the overall experience of both patients and ambulance staff, as well as use descriptive statistics and comparisons between responder groups (e.g. ambulance staff vs patients; paramedics vs emergency medical technicians), where appropriate.  WP4 Qualitative: Qualitative data analysis will involve thematic coding of collected data (i.e. framework analysis) and the audio recorded data (transcribed verbatim) will be entered into NVivo 11 qualitative data analysis software to facilitate analysis. We will use the Medical Research Council guidance^2^ on process evaluations of complex interventions as a framework to guide the analysis and synthesis of our data, complemented by inductive codes to ensure all aspects of the participants’ experience are fully captured. Accordingly, our framework will be developed based on the following key components and functions of a process evaluation: description of the intervention and its causal assumptions; the implementation process (how delivery was achieved, training, resources, etc.) and what was delivered (fidelity, adaptations, etc.); the mechanisms of impact (participant responses to and interactions with the intervention, mediators, unexpected pathways and consequences), context (contextual factors that shape theories of how the intervention works and that affect the implementation, intervention mechanisms, etc.); and outcomes.  WP5 Integration: We will integrate qualitative and quantitative data to explain the findings from the stepped wedge and economic evaluation using the interview and survey findings. |

# KEY WORDS

Ambulance calls; diabetes; hypoglycaemia; intervention; leaflet; process evaluation.

**Table of Contents**

[SIGNATURE PAGE 2](#_Toc523838056)

[STUDY/TRIAL CONTACTS 3](#_Toc523838057)

[FUNDER DETAILS 4](#_Toc523838058)

[STUDY SUMMARY 5](#_Toc523838059)

[KEY WORDS 8](#_Toc523838060)

[LIST OF ABBREVIATIONS 11](#_Toc523838061)

[STUDY MANAGEMENT 12](#_Toc523838062)

[ROLE OF STUDY SPONSOR AND FUNDER 12](#_Toc523838063)

[STUDY MANAGEMENT COMMITTEES 12](#_Toc523838064)

[Study Steering/Management Group 12](#_Toc523838067)

[STUDY BACKGROUND and RATIONALE 12](#_Toc523838068)

[STUDY OBJECTIVES AND PURPOSE 14](#_Toc523838069)

[PURPOSE 14](#_Toc523838070)

[PRIMARY OBJECTIVE 14](#_Toc523838071)

[SECONDARY OBJECTIVE(S) 14](#_Toc523838072)

[OUTCOME MEASURES/ENDPOINTS 14](#_Toc523838073)

[PRIMARY OUTCOME MEASURE/ENDPOINT 14](#_Toc523838074)

[SECONDARY ENDPOINTS/OUTCOMES 14](#_Toc523838075)

POSSIBLE UNINTENDED OUTCOMES [15](#_Toc523838077)

[STUDY DESIGN 15](#_Toc523838078)

[DATA ANALYSIS 1](#_Toc523838079)6

[STUDY SETTING 17](#_Toc523838080)

[SELECTION OF PARTICIPANTS 1](#_Toc523838081)7

[ELIGIBILITY CRITERIA 1](#_Toc523838082)7

[Inclusion Criteria 1](#_Toc523838083)7

[Exclusion Criteria 1](#_Toc523838084)7

[Sampling 1](#_Toc523838085)8

[Size of sample 18](#_Toc523838086)

[RECRUITMENT 19](#_Toc523838088)

[Participant Payment 20](#_Toc523838089)

[CONSENT 20](#_Toc523838090)

[**STUDY PROCEDURES/REGIMEN** 21](#_Toc523838091)

[**STUDY FLOWCHART** 21](#_Toc523838092)

[**RANDOMISATION AND BLINDING** 22](#_Toc523838093)

[**STUDY REGIMEN** 22](#_Toc523838094)

[**WITHDRAWAL** 23](#_Toc523838096)

[**ETHICAL AND REGULATORY CONSIDERATIONS** 23](#_Toc523838097)

[ASSESSMENT AND MANAGEMENT OF RISK 23](#_Toc523838098)

[ETHICS REVIEW AND COMPLIANCE 24](#_Toc523838099)

[PEER REVIEW 25](#_Toc523838100)

[PUBLIC & PATIENT INVOLVEMENT 25](#_Toc523838101)

[PROTOCOL COMPLIANCE 26](#_Toc523838102)

[DATA PROTECTION AND PATIENT CONFIDENTIALITY 26](#_Toc523838103)

[INDEMNITY 26](#_Toc523838104)

[ACCESS TO THE FINAL DATASET 26](#_Toc523838105)

[DISSEMINATION POLICY 27](#_Toc523838106)

[Authorship eligibility guidelines and any intended use of professional writers 27](#_Toc523838107)

[REFERENCES 2](#_Toc523838108)8

# LIST OF ABBREVIATIONS

AE Adverse Event

CF Consent Form

CI Chief Investigator

CRF Case Report Form

EMAS East Midlands Ambulance Service NHS Trust

GCP Good Clinical Practice

HRA Health Research Authority

ICF Informed Consent Form

ISF Investigator Site File (This forms part of the TMF)

ISRCTN International Standard Randomised Controlled Trials Number

LIH Lincoln Institute for Health

NHS R&D National Health Service Research & Development

PI Principal Investigator

PIS Participant Information Sheet

PPI Public and Patient Involvement

REC Research Ethics Committee

SAE Serious Adverse Event

SOP Standard Operating Procedure

TMF Trial Master File

UoL University of Lincoln

# STUDY MANAGEMENT

### ROLE OF STUDY SPONSOR AND FUNDER

The sponsor of the study is the University of Lincoln.

The Chief Investigator has overall responsibility for the study and shall oversee all study management.

## STUDY MANAGEMENT COMMITTEES

### Study Steering/Management Group

The Study Steering Committee shall meet every three months to ensure all practical details of the study are progressing and working well.

Academic team members conducting the study will meet monthly to discuss operational matters, either face-to-face or via teleconference.

# STUDY BACKGROUND and RATIONALE

Diabetes is increasing in prevalence, now affecting 5% of UK adults^3^. Efforts to improve diabetes control, using insulin and drugs to reduce complications, have led to increased rates of hypoglycaemia in people with diabetes^4^. Interruption of glucose supply to the brain causes cerebral dysfunction which, unless corrected promptly, can cause cognitive impairment, confusion, coma and, if prolonged, death.

Hypoglycaemia, a common adverse complication of diabetes treatment^5^, is often managed by patients themselves or their relatives, but severe hypoglycaemia (defined as cognitive impairment severe enough to require external assistance for recovery) frequently requires an ambulance resulting in Emergency Department attendance or hospital admission^6^. Severe hypoglycaemia constitutes around 1% of 1.7 million emergency ambulance calls in England and Scotland resulting in 100 thousand paramedic attendances and estimated costs in England of £13.6 million annually^7^.

Hypoglycaemia is associated with adverse consequences including higher mortality, morbidity and health service costs due to hospital attendance/admission^8 9 10^. It is also frightening for both patients and relatives and impairs quality of life and productivity, leading to poor long term control^11 12^.

A common accompaniment of insulin treated diabetes is impaired awareness (affecting around 25% of adults with type 1 and 10% of type 2 diabetes) when people experience reduced symptoms without having or recognising warning signs of impending hypoglycaemia. It increases the chance of a subsequent severe hypoglycaemic event by threefold^13^. Impaired awareness is more common in those who call an ambulance for hypoglycaemia with around half of type 1 diabetes individuals displaying features of reduced awareness^14^.

A systematic review highlighted that those with hypoglycaemia attended by ambulance services frequently have recurrent episodes, do not attend primary care when advised to do so by ambulance staff, but often require changes in therapy to prevent further episodes^15^. Recurrent hypoglycaemic episodes following ambulance calls are estimated to occur in 7.7% at 3 months and 17.7% at 6 months (data from Scottish Ambulance Service). The authors of the systematic review recommended development and evaluation of community referral pathways for hypoglycaemia^15^.

A systematic review and subsequent controlled before-and-after study in the Emergency Department concluded that information leaflets (booklets) can improve patients’ knowledge and satisfaction. For patients with acute conditions, leaflets can increase adherence to treatment^16 17^. Kumaran et al.^18^ also provide evidence to support the fact that patients with diabetes could be helped by providing them with well-designed educational leaflets which explain how to better manage their condition.

“Hypos can strike twice” is part of a complex quality improvement intervention involving ambulance staff providing treatment and advice to people who have had a hypoglycaemic attack to access follow-up care by the GP or specialist diabetes team, as detailed in national ambulance (JRCALC) clinical guidelines^1^, supported by the provision of a “Hypos can strike twice” booklet, which the patient can read when they are fully recovered from the cognitive and other effects of the hypoglycaemic episode.

**Description of the intervention**: The “Hypos can strike twice booklet” has a section where the paramedics record for the patient a summary of their physical condition both when the ambulance service arrives and when it departs. It also includes a section where the ambulance staff record details of the relevant treatment provided. We hope this will be useful to patients who may be confused during their hypos (and hence during the visit from the ambulance service) so that they can refer back to it later after their recovery. All patients receiving the booklet will be advised to seek further advice from their GP or local community diabetes services so that they have an opportunity to review with a health professional whether they require any changes in their usual diabetic medication/insulin. In addition to explaining to the patients what has happened to them, the booklet also provides advice about what to eat following a hypo and reminds them to avoid strenuous activities, alcohol and be cautious about driving during the first few hours of recovery. It also encourages them to undertake regular self-monitoring of their blood-glucose levels. FAQs are provided to explain in lay terms, what hypos are, the symptoms, causes, how to try to prevent them and what to do if they happen again. We hope that patients will find the booklet reassuring and serve as a useful and easy-to-read resource for them. It is hoped that this intervention will reduce the chances of the patients having further severe hypos and reduce ambulance attendances for this.

The ‘Hypo’s can strike twice’ intervention was delivered in successive county areas of East Midlands Ambulance Service NHS Trust (EMAS). Communications face-to-face, email and information publicised on an electronic bulletin (E-news), twitter and Facebook, advise staff to implement the booklet when visiting patients with diabetes who have experienced hypoglycaemia. Reminders are also provided to staff to complete the care bundle for hypoglycaemia and to refer patients to an appropriate healthcare professional following an episode of hypoglycaemia.

The intervention was rolled out by division (county) in EMAS in the following steps:

Step 1: October/November 2018, Leicester and Northamptonshire.

Step 2: January/February 2019, Nottinghamshire and Derbyshire.

Step 3: April/May 2019, Lincolnshire.

This study will evaluate the effect of implementing an ambulance clinician delivered hypoglycaemia intervention (‘Hypos can strike twice’) on repeat ambulance calls, attendances and transports to hospital and also explore how the intervention was delivered, the impact on staff and patients, the wider context in which it was delivered, and the opportunities and potential for scaling up this intervention more widely.

# STUDY OBJECTIVES AND PURPOSE

## PURPOSE

Aim: To evaluate the effect of implementing the ‘Hypo’s can strike twice’ intervention to patients with diabetes and hypoglycaemia attended by EMAS in the East Midlands region. “Hypos can strike twice” is a complex intervention involving ambulance staff providing treatment and advice to people who have had a hypoglycaemic attack to access follow-up care by the GP or specialist diabetes team as detailed in national ambulance (JRCALC) clinical guidelines^1^ supported by the provision of a “Hypos can strike twice” booklet which the patient can read when they are fully recovered from the cognitive and other effects of the hypoglycaemic episode.

## PRIMARY OBJECTIVE

To investigate the effect of implementing the ‘Hypo’s can strike twice’ on repeat ambulance calls and attendances for hypoglycaemia.

## SECONDARY OBJECTIVES

To investigate the effect of implementing the ‘Hypo’s can strike twice’ on:

1. Recorded referrals to an appropriate healthcare professional (e.g. GP, nurse).
2. Completed care bundle for hypoglycaemia (proportion of patients with all of blood glucose recorded before treatment, blood glucose recorded after treatment and treatment given for hypoglycaemia.
3. Costs of implementation ‘Hypo’s can strike twice’ intervention vs costs of health service resource use.
4. To conduct a process evaluation to explore how ‘Hypo’s can strike twice’ exerts its effects and can be scaled up if effective by understanding implementation, mechanisms of impact, and contextual factors using interviews and surveys.

# OUTCOME MEASURES/ENDPOINTS

## PRIMARY OUTCOME MEASURE/ENDPOINT

Repeat ambulance calls and attendances for hypoglycaemia within 14 days.

## SECONDARY ENDPOINTS/OUTCOMES

1. Repeat ambulance calls and attendance for hypoglycaemia within 30 and 90 days.
2. Recorded referral to an appropriate healthcare professional (e.g. GP, nurse).
3. Completed care bundle for hypoglycaemia (proportion of patients with all of blood glucose recorded before treatment, blood glucose recorded after treatment and treatment given for hypoglycaemia.
4. Costs of implementation of ‘Hypo’s can strike twice’.
5. Process evaluation to explore how ‘Hypo’s can strike twice’ exerts its effects and can be scaled up if effective by understanding implementation, mechanisms of impact, and contextual factors using interviews and surveys.

# Possible unintended outcomes

Possible unintended outcomes of the intervention may include poorer control of diabetes with a greater risk of microvascular complications but this has to be balanced with the risk of hypoglycaemia and patients’ preferences for treatment and quality of life.

# STUDY DESIGN

Non-randomised stepped wedge design with mixed methods process evaluation.

Work package (WP) 1 Stepped wedge: We will use routine anonymised data from routine call-and-dispatch and clinical records data from EMAS for 12 months before the intervention was first introduced (September 2017) to at least 6 months after the final step of the introduction in April 2019, i.e. October 2019. We will include call categories, timings, geographical location, together with demographic (age, sex) and clinical information, physiological measurements taken, treatments given, and whether the patient was referred to community services (general practitioner, community diabetes nurse or alternative) for their diabetes or transported to hospital by ambulance. Hypoglycaemia is recorded in electronic clinical records under ‘chief complaint’ or impression’ which will enable us to identify incidents of patients presenting with hypoglycaemia. Individual patients will be assigned a unique (non-personally identifiable) number in the anonymised dataset provided to the university which will enable us to identify repeat attendances for an individual patient.

WP2 Health economics: We will use a cost-consequences approach to estimate the costs of the intervention in relation to its effects. Total costs will be estimated, from an NHS perspective, using unit costs from NHS Reference Costs, Emergency Services Reference Costs, and other relevant costs, multiplied by resource units.

WP3 Survey: We will use a self-administered survey to understand implementation, mechanisms of impact, and contextual factors. We will use random sampling to select patients with hypoglycaemia who were attended stratified by geographical area, from the ambulance service user records and we will send them questionnaires by post (with a stamped addressed envelope for return to the University of Lincoln) or offer them the option to complete the questionnaire on-line, according to their preference. We will conduct an online survey to all front-line clinicians to assess attitudes, perceptions and behaviour towards the intervention including the booklet. We will also conduct a self-administered postal questionnaire to patients using a Patient Reported Experience Measure, previously developed and validated by Siriwardena and colleagues^19 20^. The sample size calculation for the survey is included below.

WP4 Qualitative: We will conduct semi-structured interviews, as part of the process evaluation, to explore whether the introduction of the ‘Hypo’s can strike twice’ intervention was considered beneficial by participants, how acceptable the intervention was, and whether it was associated with any unanticipated issues. Through the use of interviews we will also assess the fidelity and quality of the implementation, as well as explore causal mechanisms and contextual factors. We will answer three main questions: What is implemented and how? How does the delivered intervention produce change? How does context affect implementation and outcomes?

## We will interview a purposive sample of ambulance clinicians in EMAS who delivered the intervention and patients with hypoglycaemia who were attended in the geographical areas following introduction of the intervention using maximum variation in characteristics such as practitioner age, sex and geographical region and patient age, sex, and geographical region. For the patient sample we will also include patients who did or did not experience a recurrent hypoglycaemic episode as part of the intervention. We will include approximately 10-15 staff and 10-15 patients until we achieve theoretical saturation.

## DATA ANALYSIS

WP1 Stepped wedge: Dispatch data will be linked with clinical data using a unique job number. People included will be identified and tracked for repeat attendances. In the stepped wedge design, the intervention and control observation periods take the place of the treatment and control groups in a parallel trial and the distribution of results across unexposed observation periods is compared with that across the exposed observation periods. There are a small number of steps and so characteristics of the groups in the wedge can be compared by group, including number, the average cluster size, cluster characteristics, and important patient characteristics.

Following an intention-to-treat principle, clusters will be analysed according to their crossover time irrespective of whether crossover was achieved at the desired time. Under the stepped wedge design the evaluation happens over a period of time, during which the number of clusters exposed to the intervention gradually increases. This means that unexposed observations will, on average, be from an earlier calendar time than exposed observations. Additionally, policy and service delivery changes may occur. Calendar time is associated with both the exposure to the intervention and also possibly the outcome, and so is a potential confounder and will be adjusted for in the analysis.

A linear mixed model will be fitted adjusting for the systematically different observation periods and for clustering in the data. Time is included as a fixed effect and a random effect for cluster. The design also allows examination of the impact of the intervention changing over time once it is introduced into a cluster.

WP2 Health economics: We will use a cost consequences model, estimating costs from NHS Reference Costs to evaluate the costs of the intervention in relation to its effects. Key cost drivers associated with repeat hypoglycaemic episodes will be modelled.

WP3 Survey: We will analyse the overall experience of both patients and ambulance staff, as well as use descriptive statistics and comparisons between responder groups (e.g. ambulance staff vs patients; paramedics vs emergency medical technicians), where appropriate.

WP4 Qualitative: Qualitative data analysis will involve thematic coding of collected data (i.e. framework analysis) and the audio recorded data (transcribed verbatim) will be entered into NVivo 12 qualitative data analysis software to facilitate analysis. We will use the Medical Research Council guidance on process evaluations of complex interventions^2^ as a framework to guide the analysis and synthesis of our data, complemented by inductive codes to ensure all aspects of the participants’ experience are fully captured. Accordingly, our framework will be developed based on the following key components and functions of a process evaluation: description of the intervention and its causal assumptions; the implementation process (how delivery was achieved, training, resources, etc.) and what was delivered (fidelity, adaptations, etc.); the mechanisms of impact (participant responses to and interactions with the intervention, mediators, unexpected pathways and consequences), context (contextual factors that shape theories of how the intervention works and that affect the implementation, intervention mechanisms, etc.); and outcomes.

WP5 Integration: We will integrate qualitative and quantitative data to explain the findings from the stepped wedge and economic evaluation using the interview and survey findings.

## STUDY SETTING

This is a single centre study. All participants will be recruited from routine call-and-dispatch and clinical records data from East Midlands Ambulance Service NHS Trust.

# SELECTION OF PARTICIPANTS

## ELIGIBILITY CRITERIA

Ensure consistency with A15 IRAS

### Inclusion Criteria

**Patients**

Young adults aged 16-17 years and adults aged 18 years+ with diabetes experiencing hypoglycaemia needing an ambulance service response. Hypoglycaemia for this study was identified as a ‘clinical impression’ of ‘hypoglycaemia’ or ‘diabetic problem’. Hypoglycaemia may also be identified according to ambulance guidelines^1^ as any blood glucose less than 4mmol/L.

**Clinicians**

Ambulance staff providing treatment and advice to people who have had a hypoglycaemic attack.

### Exclusion Criteria

**Patients**

Children under the age of 16 years.

**Clinicians**

Ambulance staff providing treatment and advice for incidents other than hypoglycaemia.

### Sampling

### Size of sample

WP1 and 2 Stepped wedge (non-randomised control design):

3 clusters at county level (1.Leicestershire+Northamptonshire; 2.Derbyshire+Nottinghamshire; 3.Lincolnshire) implementing intervention at each time step lasting around 3 months, so 12 data collection periods (one collection per cluster per time step).

Primary outcome: Proportion of repeat ambulance calls and attendances for hypoglycaemia within 14 days.

Significance level = 0.05

Intraclass coefficient = 0.1 (conservative estimate)

The number of hypo calls per time period (3 months) per county group was estimated from routine data from EMAS for years 2012-2017 (6 years).

| **County group** | **Frequency** | **Percentage** |
| --- | --- | --- |
| Leicestershire | 639,811 | 19.39 |
| Northamptonshire | 449,916 | 13.63 |
| Derbyshire | 662,745 | 20.08 |
| Nottinghamshire | 773,066 | 23.43 |
| Lincolnshire | 774,128 | 23.46 |
| Total | 3,299,972 | 100.00 |

Number of hypos expected every 3 months = 1% of attendances

Leicestershire Northamptonshire = (639811+449916)*0.01*(1/24) = 454

Derbyshire Nottinghamshire = (662745+773066)*0.01*(1/24) = 598

Lincolnshire = (774128)*0.01*(1/24) = 323

Proportion pre-intervention with a repeat attendance = 0.1

Power is fixed at 80%.

The detectable difference is a reduction of repeat attendances of 5% (i.e. from 10% to 5%).

WP3 Survey: We will survey all front-line staff. We will survey patients who received the intervention based on a response rate of 20% from a previously published study using the Ambulance Patient Reported Experience Measure.^19 20^ In this study the experience measure included 3 components AmbCallScore (mean 62.37, standard deviation 26.7), AmbCareScore (mean 85.6, standard deviation 16.4) and AmbLeaveScore (mean 73.77, standard deviation 21.6) Assuming the proportion of sample with the expected outcome: 0.50, margin of error: +/- 10.0%, confidence level: 95.0%, the estimated sample size required is 96. The estimate adjusted for a 20% response rate is 447. This means sampling from 447 we anticipate an estimate of each of the scores that is within 10% of the true value.

WP4 Qualitative; The qualitative sample of 10-15 staff and 10-15 patients is based on previous experience of the sample required to achieve theoretical saturation.

## RECRUITMENT

To identify potential participants, we will use routine anonymised data from routine call-and-dispatch and clinical records data from EMAS for 12 months before the intervention was first introduced (September 2017) to at least 6 months after the final step of the introduction in April 2019, i.e. October 2019. The data will be obtained and pseudo-anonymised by a member of the team at EMAS. The final dataset for this study will only be accessible to the Chief Investigator, the Clinical Database Manager, the Research Assistant(s), the Health Economist(s), and the Statistician(s).

Eligible ambulance staff members will receive an email, from a member of the team at EMAS, explaining the purpose of the study and what it will involve and inviting them to participate in the study. The email will also include a PIS and a link to the online survey. Participants will be asked to give their consent online, as part of the online questionnaire, and unless consenting to all statements of the consent form, they will not be able to continue to the questionnaire.

Members of the team at EMAS will send (by post) a study pack to eligible patients, including a study PIS, a consent form, a paper copy of the questionnaire and a pre-paid envelope, so that they can return their completed questionnaires. The PIS will inform patients of all study procedures.

Participants (both ambulance staff members and patients) will be offered the option of having a chat with one of the researchers, so that they can discuss the study and ask any possible questions they may have.

Participants will also have the opportunity to express their interest (as part of completing the questionnaires) in having an interview at a later date with one of the research team members. Following this, a member of the research team will contact potential participants by telephone to verify study eligibility, and answer any questions the participants may have. Once verbal consent has been received from participants, the researchers will schedule a face-to-face or telephone interview, including obtaining written informed consent.

Under the General Data Protection Regulation (GDPR), East Midlands Ambulance Service NHS Trust (EMAS) is the Data Controller for any personal information it processes. When EMAS shares patient information for research purposes, they ask for patient consent before doing so. Even if patients do consent, they are allowed to withdraw this consent at any time if they change their mind. EMAS always removes any information that may identify patients if it is not necessary. Accordingly, statistical information, requiring anonymised data, will always be used whenever possible.

If needed, the usual EMAS interpreter and translator services will be available to assist with discussion of the trial, the participant information sheets, and consent forms, but the consent forms and information sheets will not be available printed in other languages.

It will be explained to the potential participant that entry into the trial is entirely voluntary and that their treatment and care will not be affected by their decision. It will also be explained that they can withdraw at any time but attempts will be made to avoid this occurrence. In the event of their withdrawal it will be explained that their data collected so far may not be erased in accordance with the University’s Research Privacy Notice and information given in the Participant Information Sheet and we will seek consent to use the data in the final analyses where appropriate.

### Participant Payment

Participants will not be paid to participate in the intervention. All data collection is to occur online or in the homes of patients or via telephone, hence, no visits in excess of usual care are expected.

## CONSENT

All participants shall provide written informed consent. The ICF will be signed and dated by the participant before they enter the study.

Ambulance staff members completing the online questionnaires will consent online, prior to being able to access the questionnaire. Patients will be sent consent forms by post as part of their study packs and they will need to return their signed consent forms via a pre-paid envelope.

For participants wishing to have an interview, a member of the research team will obtain written informed consent prior to the interview taking place.

The process for obtaining participant informed consent or assent will be in accordance with the REC guidance, and Good Clinical Practice (GCP) and any other regulatory requirements that might be introduced. The investigator or their nominee and the participant or other legally authorised representative shall both sign and date the Informed Consent Form before the person can participate in the study.

One copy of the ICF will be kept by the participant and one will be kept by the Investigator.

Should there be any subsequent amendment to the final protocol, which might affect a participant’s participation in the trial, continuing consent will be obtained using an amended Consent form which will be signed by the participant.

**STUDY PROCEDURES/REGIMEN**

**STUDY FLOWCHART**

Recruitment

Pre-award

Figure 1. Schematic overview of the study.

**RANDOMISATION AND BLINDING**

This study will not involve randomisation or blinding of participants or study personnel.

**STUDY REGIMEN**

The ‘Hypo’s can strike twice’ intervention was delivered in successive county areas of East Midlands Ambulance Service NHS Trust (EMAS).

**Effectiveness of the intervention**

We will measure the effect of ‘Hypos can strike twice’ intervention on repeat ambulance calls and transportation to the hospital Emergency Departments. We will use routine anonymised data from routine call-and-dispatch and clinical records data from EMAS for 12 months before the intervention was first introduced (September 2017) to at least 6 months after the final step of the introduction in April 2019, i.e. October 2019. Dispatch data will be linked with clinical data using a unique job number. People included will be identified and tracked for repeat attendances and referrals to an appropriate healthcare professional (e.g. GP, nurse); our primary outcome will be the proportion of repeat ambulance calls and attendances for hypoglycaemia within 14 days. Repeat ambulance calls and attendance for hypoglycaemia within 30 and 90 days will be a secondary outcome.

**Cost-effectiveness of the intervention**

We will use a cost-consequences model, estimating costs from NHS Reference Costs to evaluate the costs of the intervention in relation to its effects. Total costs associated with repeat hypoglycaemic episodes will be estimated, from an NHS perspective, using unit costs from NHS Reference Costs, Emergency Services Reference Costs, and other relevant costs, multiplied by resource units.

**Process evaluation**

We will conduct a process evaluation using surveys and interviews of ambulance staff and patients to explore how ‘Hypo’s can strike twice’ exerts its effects and can be scaled up, if effective, by understanding implementation, mechanisms of impact, and contextual factors.

*Survey*

We will use a self-administered survey to understand implementation, mechanisms of impact, and contextual factors. We will use random sampling to select patients with hypoglycaemia who were attended, stratified by geographical area, from the ambulance service user records and we will send them questionnaires by post (with a stamped addressed envelope for return to the University of Lincoln) or offer them the option to complete the questionnaire on-line, according to their preference. We will conduct an online survey to all front-line clinicians to assess attitudes, perceptions and behaviour towards the intervention including the booklet. We will also conduct a self-administered postal questionnaire to patients using a Patient Reported Experience Measure^19 20^. We will survey all front-line staff and approximately 447 patients.

We will analyse the overall experience of both patients and ambulance staff, as well as use descriptive statistics and comparisons between responder groups (e.g. ambulance staff vs patients; paramedics vs emergency medical technicians), where appropriate.

## *Qualitative study*

We will interview a purposive sample (approximately 10-15 staff members) of ambulance clinicians in EMAS, who delivered the intervention, and patients (approximately 10-15) with hypoglycaemia who were attended in the geographical areas following introduction of the intervention. For the patient sample we will also include patients who did or did not experience a recurrent hypoglycaemic episode as part of the intervention.

*Integration*

We will integrate qualitative and quantitative data to explain the findings from the stepped wedge and economic evaluation, using the interview and survey findings.

## WITHDRAWAL

Participants may be withdrawn from the trial either at their own request or at the discretion of the Investigator. The participants will be made aware that this will not affect their future care. Participants will be made aware (via the information sheet and consent form) that should they withdraw the data collected to date may not be erased in accordance with the University’s Research Privacy Notice and information given in the Participant Information Sheet and may still be used in the final analysis.

# ETHICAL AND REGULATORY CONSIDERATIONS

## ASSESSMENT AND MANAGEMENT OF RISK

Individual participant medical information obtained as a result of this study are considered confidential and disclosure to third parties is prohibited with the exceptions noted. Medical information may be given to the participant’s medical team and all appropriate medical personnel responsible for the participant’s welfare.

If information is disclosed during the study that could pose a risk of harm to the participant or others, the researcher will discuss this with the CI and where appropriate report accordingly.

## ADVERSE EVENTS

There are no serious adverse events anticipated with participating in this study.

Possible unintended outcomes of the intervention may include poorer control of diabetes with a greater risk of microvascular complications, but this has to be balanced with the risk of hypoglycaemia and patients’ preferences for treatment and quality of life.

## ETHICS REVIEW AND COMPLIANCE

The study shall not commence until the study protocol, information sheets and consent forms have been reviewed and approved from a Research Ethics Committee and relevant NHS/Social Care permission is obtained.

The sponsor will be responsible for deciding whether amendments are substantial and non-substantial in collaboration with the Chief Investigator.

Where an amendment is required to study documentation that required REC approval, changes will not be implemented until REC approval and HRA categorisation is received. Where an amendment requires local approval this shall be sought prior to the amendment be implemented at each site in accordance with the categorisation given on the HRA approval letter.

Should an amendment be required to eliminate an apparent immediate hazard to participants this may be implemented immediately and the REC/HRA and R&D will be notified as soon as possible.

Minor amendments for logistical or administrative purposes may be implemented immediately

Amendments will be logged on the Sponsor’s Study Amendment Log and stored in the Trial Master/Site File(s).

Annual Progress Reports shall be submitted to the REC within 30 days of the anniversary date on which the favourable opinion was given – until the end of the study.

A final report shall (where possible) be submitted to the REC within one year after the end of the study.

If the study is terminated prematurely the CI will notify the REC, including the reasons for premature termination.

## PEER REVIEW

This protocol has been internally and externally reviewed. Internal review has been undertaken to assess the ethical aspects of this study and advise on scientific quality of the study. External review was taken as part of the funding application to NIHR Applied Research Collaboration (ARC) East Midlands.

### PUBLIC & PATIENT INVOLVEMENT

Patients and the public were involved in the development of this project through a meeting with the South Lincolnshire Diabetes Group in 2018 and two meetings which took place in February and July 2019 with the Healthier Ageing Patient and Public Involvement (HAPPI) Group at the University of Lincoln.

The South Lincolnshire diabetes service user group were concerned about hypoglycaemic episodes (hypos) and felt this was a very important problem. What mattered to them was good care to reduce further hypos, increase specialist follow up, improve experience of care, and reduce unnecessary hospital admissions and costs. The HAPPI group (which includes people with diabetes and their relatives) support ambulance based research. They felt this was an important problem for ambulance services. Members offered advice on including patients and where appropriate their carers/relatives.

Members of all these groups were keen to support the study and join the study team and will be involved as members of the steering group and through discussions with the groups to:

- ensure accessible and understandable language in patient information;

- clarify the importance for patients of reducing severe hypos and unnecessary hospitalisation through appropriate community support;

- highlight important outcome measures such as reduced hypos and good care experience;

- explore the best way to ensure effective ongoing contribution of PPI to the project through steering group membership and regular discussion with both groups;

- highlight PPI contribution for the development of patient resource and dissemination;

- identify future training and ongoing support needs for PPI.

We will benefit from PPI input to the study and its steering group from Keith Spurr (chair) and South Lincolnshire Diabetes Group, Amanda Brewster (chair), Pauline Mountain and other members of the Healthier Aging PPI (HAPPI) group (University of Lincoln).

Ensure consistency with A14 IRAS.

## PROTOCOL COMPLIANCE

Accidental protocol deviations may occur at any time. Accidental protocol deviations will be adequately documented on the relevant forms and reported to the Chief Investigator and Sponsor immediately.

Deviations from the protocol which are found to frequently recur are not acceptable, these will require immediate action and could potentially be classified as a serious breach.

# DATA PROTECTION AND PATIENT CONFIDENTIALITY

All study staff and investigators will comply with the principles of the Data Protection Act (2018) in protecting the rights of study participants with regards to the collection, storage, processing and disclosure of personal information and will uphold the Act’s/Regulations core principles.

Each participant will be assigned a study identity number, for use on CRFs other trial documents and the electronic database.

Personal data, research data and the linking code will be stored in separate locations. When stored electronically, this will include using encrypted digital files within password protected folders and storage media. Personal information shall be stored separately to research data and will be kept secure, and maintained.

Personal data will be stored for 5 years following the end of the study, so that the Chief Investigator may provide participants with a summary of the research (should they wish to receive a copy).

Data generated as a result of this study will be available for inspection on request by the participating physicians, the University of Lincoln representatives, the REC, local R&D Departments and the regulatory authorities.

# INDEMNITY

Insurance and indemnity for trial participants and trial staff is covered within the NHS Indemnity Arrangements for clinical negligence claims in the NHS, issued under cover of HSG (96)48. There are no special compensation arrangements, but trial participants may have recourse through the NHS complaints procedures.

The University of Lincoln as research Sponsor indemnifies its staff, research participants and research protocols with both public liability insurance and clinical trials insurance.

## ACCESS TO THE FINAL DATASET

The final dataset for this study will only be accessible to the Chief Investigator, the Clinical Database Manager, the Research Assistant(s), the Health Economist(s), and the Statistician(s).

# DISSEMINATION POLICY

The data custodian will be the Chief Investigator on behalf of the University of Lincoln.

Plans are made to disseminate the findings in peer-reviewed journals, major EMS and/or diabetes conferences (oral and/or poster presentations) and as written reports to the University of Lincoln, East Midlands Ambulance Service NHS Trust, and the NIHR Applied Research Collaboration (ARC) East Midlands. Dissemination will be tailored to the following audiences: Academic, ambulance services, community trusts, general practice, public health, patients and the public, diabetes charities, and diabetes service user groups.

### Authorship eligibility guidelines and any intended use of professional writers

All authors of peer-reviewed articles from this study will meet the four following criteria for authorship in line with The International Committee of Medical Journal Editors: provide substantial contribution to the conception or design of the work, or the acquisition, analysis or interpretation of data for the work; contribute to draft copies of work and revising it critically for important intellectual content; review and approve the final version for publication; and agree to be accountable for all aspects of the work in ensuring that questions related to the accuracy or integrity of any part of the work are appropriately investigated and resolved.

# REFERENCES

1. Joint Royal Colleges Ambulance Liaison Committee (JRCALC), Association of Ambulance Chief Executives (2019) JRCALC Clinical Guidelines 2019. Bridgwater: Class Professional Publishing.
2. Moore GF, Audrey S, Barker M, et al. Process evaluation of complex interventions: Medical Research Council guidance. BMJ 2015;350:h1258. doi: 10.1136/bmj.h1258
3. Holman N, Young B, Gadsby R. Current prevalence of Type 1 and Type 2 diabetes in adults and children in the UK. Diabet Med 2015;32(9):1119-20. doi: 10.1111/dme.12791
4. Boussageon R, Bejan-Angoulvant T, Saadatian-Elahi M, et al. Effect of intensive glucose lowering treatment on all cause mortality, cardiovascular death, and microvascular events in type 2 diabetes: meta-analysis of randomised controlled trials. BMJ 2011;343:d4169. doi: 10.1136/bmj.d4169
5. Barendse S, Singh H, Frier BM, et al. The impact of hypoglycaemia on quality of life and related patient-reported outcomes in Type 2 diabetes: a narrative review. Diabet Med 2012;29(3):293-302. doi: 10.1111/j.1464-5491.2011.03416.x
6. Heller SR, Frier BM, Herslov ML, et al. Severe hypoglycaemia in adults with insulin-treated diabetes: impact on healthcare resources. Diabet Med 2016;33(4):471-7. doi: 10.1111/dme.12844
7. Khunti K, Fisher H, Paul S, et al. Severe hypoglycaemia requiring emergency medical assistance by ambulance services in the East Midlands: a retrospective study. Prim Care Diabetes 2013;7(2):159-65. doi: 10.1016/j.pcd.2013.01.001
8. Hemmingsen B, Lund SS, Gluud C, et al. Intensive glycaemic control for patients with type 2 diabetes: systematic review with meta-analysis and trial sequential analysis of randomised clinical trials. BMJ 2011;343:d6898. doi: 10.1136/bmj.d6898
9. Goto A, Arah OA, Goto M, et al. Severe hypoglycaemia and cardiovascular disease: systematic review and meta-analysis with bias analysis. BMJ 2013;347:f4533. doi: 10.1136/bmj.f4533
10. Yeh JS, Sung SH, Huang HM, et al. Hypoglycemia and risk of vascular events and mortality: a systematic review and meta-analysis. Acta Diabetol 2016;53(3):377-92. doi: 10.1007/s00592-015-0803-3
11. Currie CJ, Morgan CL, Poole CD, et al. Multivariate models of health-related utility and the fear of hypoglycaemia in people with diabetes. Curr Med Res Opin 2006;22(8):1523-34. doi: 10.1185/030079906X115757
12. Davis RE, Morrissey M, Peters JR, et al. Impact of hypoglycaemia on quality of life and productivity in type 1 and type 2 diabetes. Curr Med Res Opin 2005;21(9):1477-83. doi: 10.1185/030079905X61929
13. Schopman JE, Geddes J, Frier BM. Frequency of symptomatic and asymptomatic hypoglycaemia in Type 1 diabetes: effect of impaired awareness of hypoglycaemia. Diabet Med 2011;28(3):352-5. doi: 10.1111/j.1464-5491.2010.03203.x
14. Geddes J, Schopman JE, Zammitt NN, et al. Prevalence of impaired awareness of hypoglycaemia in adults with Type 1 diabetes. Diabet Med 2008;25(4):501-4. doi: 10.1111/j.1464-5491.2008.02413.x
15. Fitzpatrick D, Duncan EA. Improving post-hypoglycaemic patient safety in the prehospital environment: a systematic review. Emerg Med J 2009;26(7):472-8. doi: 10.1136/emj.2008.062240
16. Sustersic M, Gauchet A, Foote A, et al. How best to use and evaluate Patient Information Leaflets given during a consultation: a systematic review of literature reviews. Health Expect 2017;20(4):531-42. doi: 10.1111/hex.12487 [published Online First: 2016/09/28]
17. Sustersic M, Tissot M, Tyrant J, et al. Impact of patient information leaflets on doctor-patient communication in the context of acute conditions: a prospective, controlled, before-after study in two French emergency departments. BMJ Open 2019;9(2):e024184. doi: 10.1136/bmjopen-2018-024184 [published Online First: 2019/02/23]
18. Kumaran KSGA, Palanisamy S, Rajasekaran A. Development and implementation of patient information leaflets in diabetes mellitus. Journal of Pharmaceutical Health Services Research 2010;1:85-89. doi: 10.1111/j.1759-8893.2010.00006.x
19. Siriwardena AN, Togher F, Akanuwe J, et al. Reliability and validity of an ambulance patient reported experience measure (a-prem): pilot study. EMJ 2017;34(10):e6-e6.
20. Togher F, Siriwardena AN, Windle K. Refining questionnaire items in a Patient Reported Experience Measure (PREM) designed for users of NHS ambulance services: question appraisal study. EMJ 2016;33:e5-6. doi: 10.1136/emermed-2016-206139.20
